# Chitosan nanoparticles improve physiological and biochemical responses of *Salvia abrotanoides* (Kar.) under drought stress

**DOI:** 10.1186/s12870-022-03689-4

**Published:** 2022-07-22

**Authors:** Samaneh Attaran Dowom, Zahra Karimian, Mahboubeh Mostafaei Dehnavi, Leila Samiei

**Affiliations:** 1 Department of Biology, Faculty of Sciences, Ferdowsi University of Mashhad, Mashhad, Iran; 2 Department of Ornamental plants, Research center for plant Sciences, Ferdowsi University of Mashhad, Mashhad, Iran; 3 Department of Plant Sciences, University of California, Davis, CA 95616 USA

**Keywords:** Antioxidant enzymes, Biochemical responses, Chitosan nanoparticles, Drought stress, *Salvia abrotanoides*, Water deficit

## Abstract

**Background:**

The use of organic nanoparticles to improve drought resistance and water demand characteristics in plants seems to be a promising eco-friendly strategy for water resource management in arid and semi-arid areas. This study aimed to investigate the effect of chitosan nanoparticles (CNPs) (0, 30, 60 and 90 ppm) on some physiological, biochemical, and anatomical responses of *Salvia abrotanoides* under multiple irrigation regimes (30% (severe), 50% (medium) and 100% (control) field capacity).

**Results:**

The results showed that drought stress decreases almost all biochemical parameters. However, foliar application of CNPs mitigated the effects caused by drought stress. This elicitor decreased electrolyte conductivity (35%), but improved relative water content (12.65%), total chlorophyll (63%), carotenoids (68%), phenol (23.1%), flavonoid (36.4%), soluble sugar (58%), proline (49%), protein (45.2%) in *S. abrotanoides* plants compared to the control (CNPs = 0). Furthermore, the activity of antioxidant enzymes superoxide dismutase (86%), polyphenol oxidase (72.8%), and guaiacol peroxidase (75.7%) were enhanced after CNPs treatment to reduce the effects of water deficit. Also, the CNPs led to an increase in stomatal density (5.2 and 6.6%) while decreasing stomatal aperture size (50 and 25%) and semi-closed stomata (26 and 53%) in leaves.

**Conclusion:**

The findings show that CNPs not only can considerably reduce water requirement of *S. abrotanoides* but also are able to enhance the drought tolerance ability of this plant particularly in drought-prone areas.

**Graphical abstract:**

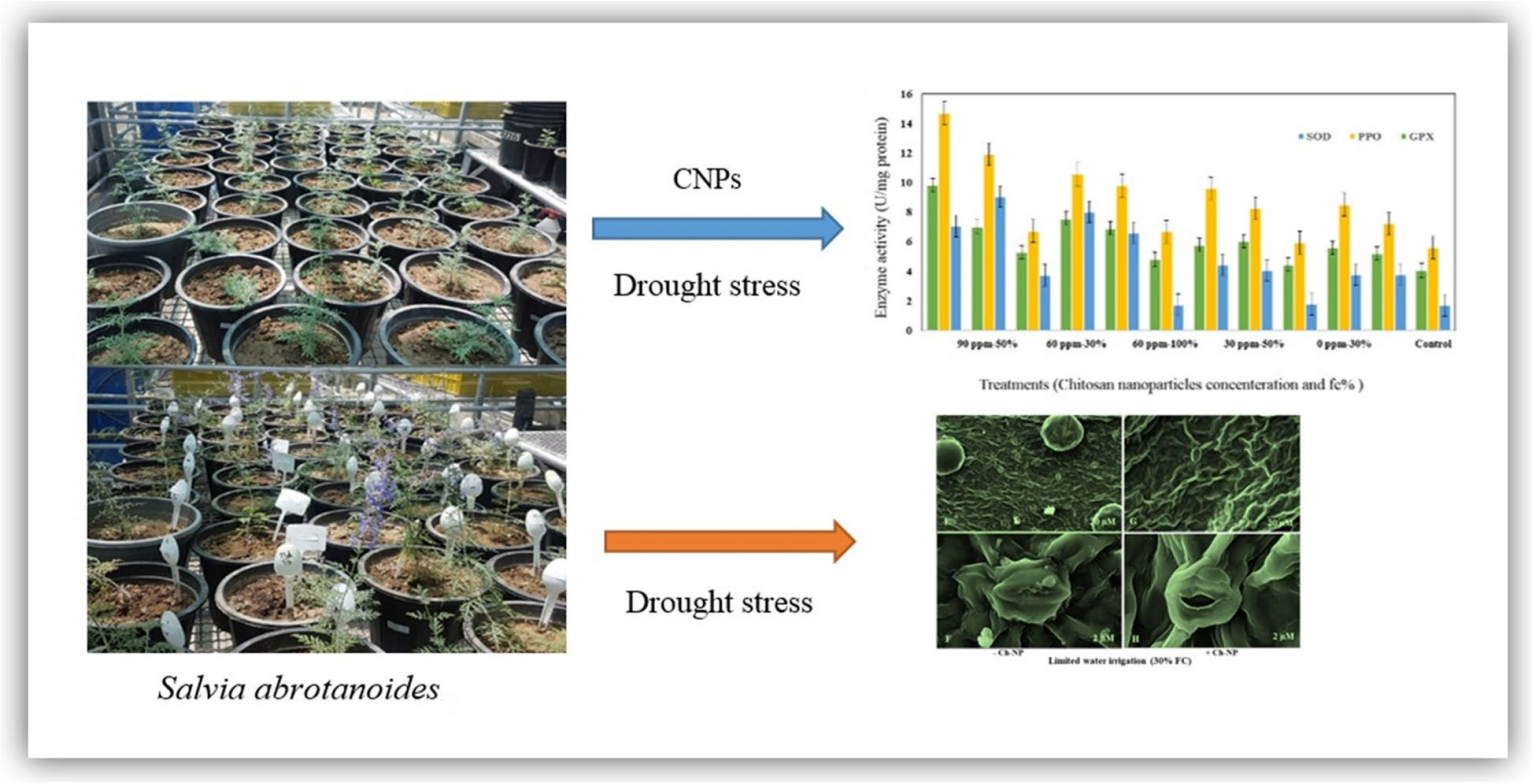

**Supplementary Information:**

The online version contains supplementary material available at 10.1186/s12870-022-03689-4.

## Highlights

• Foliar application of CNPs can improves drought tolerance.

• Leaves showed a high antioxidant enzyme activity under drought stress using CNPs.

• Biochemicals varied by drought stress levels and CNPs concentrations.

• The highest and the lowest stomatal density and aperture size, respectively were observed in the plants treated with CNPs under drought stress.

**Supplementary Information:**

The online version contains supplementary material available at 10.1186/s12870-022-03689-4.

## Background

As one of many adverse effects of climate change, prolonged droughts are a significant challenge in the twenty-first century. Forecasts show that many countries will be affected by more droughts and water scarcity issues in the coming years [1]. Droughts cause extensive damage to agriculture, urban landscape, rangelands, and forests every year. Drought stress (DS) is an inhibitor of plant growth and adversely affects crop physiology, morphology, and yield [2]. Water deficit conditions impair a plant’s physiological and biochemical processes, resulting in decreased yields [3, 4]. Drought stress generates of reactive oxygen species (ROS) and damages proteins, lipids, and nucleic acids, impairing the plant’s photosynthetic system and ATP synthesis [5]. Plants adapt a variety of physiochemical mechanisms to mitigate the adverse effects of water stress [6]. For instance, accumulating compatible solutes such as proline, carbohydrates, and amino acids mentioned cell turgidity and osmotic adjustment [7]. Reduced stomata and increased antioxidant enzyme activity are other mechanisms for ROS scavenging and mitigating DS in plants [6]. In severe DS, these mechanisms are insufficient and acquire exogenous application of some materials, including natural and organic compounds, that elevate plant resilience [7].

Chitosan is a non-toxic, non-allergenic, cost-effective, biodegradable, and eco-friendly biopolymer that serves various purposes in the agricultural, biomedical, and feed industries [8, 9]. This natural substance promotes plants’ growth and biological and non-biological resistance [10–15]. Chitosan nanoparticles (CNPs) are more effective than bulk scale normal chitosan due to their small size (less than 100 nm), high aspect ratio, and surface area [16]. They enhance plant metabolic activity and transport active chemicals more efficiently across cell membranes [17]. Although CNPs have been proven to have beneficial impacts on plant quality and productivity, there are limited reports about their ability to promote plant immune systems under abiotic stress such as drought [16].

Some studies showed that exogenous treatment of CNPs caused a significant improvement in the plant’s innate immune response through induction of the defense enzyme activity [18], an increase in the total phenolic content [14], stimulating photosynthetic rate [6], enhanced content of chlorophylls and carotenoids and mineral uptake [9], stomatal closure (ABA synthesis) [6], and induction of proline, sugars and amino acids (osmotic adjustment and turgor pressure maintenance) [19], and reduced transpiration [6]. In addition, CNPs inducing antioxidant potential and gene expression of secondary metabolites could alleviate DS in some species [6]. Soil and foliar application of CNPs on *Triticum aestivum* [20], *Catharanthus roseus* [6], *Zea mays* L. [21], and *Saccharum* spp. mitigated adverse effects of drought through the increase of the chlorophyll concentration, photosynthesis rate, and activity of antioxidant enzymes, relative water content (RWC), yield, and biomass.

*Salvia abrotanoides* (Kar.), also known as *Proveskia abrotanoides,* is a species of the family Lamiaceae native to the arid and semi-arid regions of Central and South Asia, including parts of Iran. Various phytochemical compounds include rosmarinic acid, tanshinones, and monoterpenes in the essential oils, have been identified in this species [22]. This plant is known for antioxidant, antidiabetic, anti-plasmodial, antibacterial, and anti-inflammatory properties [22–24]. *S. abrotanoides* also has significant aesthetic value due to its long flowering period, attractive flowers, unique colors, and pleasant scent. It is one of the most popular widespread species in xeriscaping and is used as an ornamental plant in the landscape of arid and semi-arid regions due to its good resistance to DS. Recently, *S. abrotanoides* has been propagated by seeds and planted in botanic gardens and green spaces in some areas [25–27].

Given the climatic and ecological conditions of the natural habitats of *S. abrotanoides*, we hypothesize that this species is an ornamental-medicinal plant with relatively good tolerance to environmental stresses, especially drought. There have been some reports about this plant’s ability to withstand water shortages and hard climate [22, 28]. Up to our knowledge, there is no information about the effect of nanoparticles on mitigating DS effect in this species. Therefore, the objective of this study was to investigate the effect of CNPs on some biochemical and physiological mechanisms involved in drought tolerance and adaptation in *S. abrotanoides*. For this purpose, physiological and biochemical parameters such as RWC, electrolyte conductivity (EC), total chlorophyll, carotenoid, phenol, flavonoid, proline, sugar, protein, and antioxidant enzymes activity, and stomatal morphology were quantified.

## Results

### Effect of DS and CNPs on relative water content (RWC) and electrolyte conductivity (EC)

**Fig. 1:**
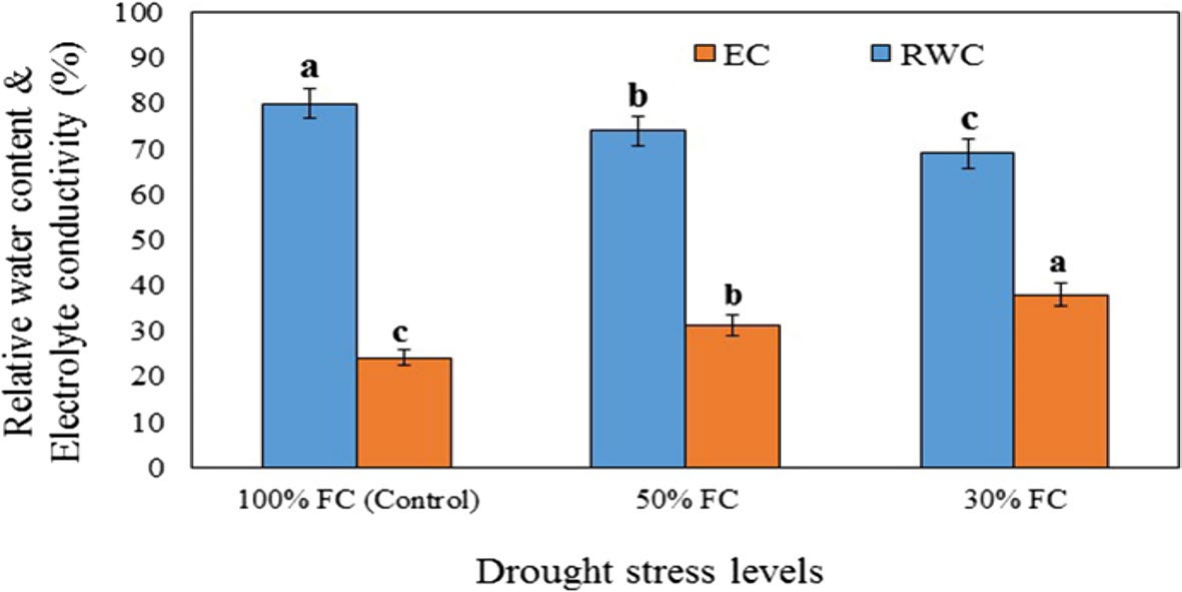
Effect of drought stress levels (30, 50, and 100% FC) on relative water content (RWC) and electrolyte conductivity (EC) in leaves of *S. abrotanoides* after 30 days. Values are means ± SD (*n* = 4). According to Duncan’s Multiple Range Test, different bar letters indicate significant differences among treatments (*P* ≤ 0.05)

Results of statistical analysis showed that both RWC and EC were significantly affected by DS (*P* ≤ 0.05) but not by CNPs or the interaction of DS and CNPs (*P* ≥ 0.05) (Table 1, Fig. 1). The comparison of means showed that the highest RWC, 82.14%, was obtained in the plants with regular irrigation (100% FC) and RWC decreased to as low as 20% when the plants were subjected to severe water stress (Fig. 1).

**Table 1:** Effect of interaction drought stress (DS) × chitosan nanoparticles (CNPs) on some biochemical and physiological traits in leaves of *S.abrotanoides*

DS×CNPs	RWC	EC	Sugar	Chl	CAR	Proline	Phenol	FLAV	Protein	SOD	PPO	GPO
**D1 × C1**	82.14^a^	19.91^a^	12.58^h^	1.02^a^	0.053^a^	10.89^f-h^	65.50^e^	0.85^a^	20.13^d^	1.71^f^	5.62^h^	4.07^g^
**D1 × C2**	80.35^a^	21.69^a^	12.88^gh^	1.16^a^	0.053^a^	8.81^h^	79.25^d^	0.85^a^	21.99^cd^	1.78^f^	5.94^h^	4.46^fg^
**D1 × C3**	79.63^a^	27.07^a^	14.95^f^	1.14^a^	0.052^a^	9.38^gh^	77.60^d^	1.00^a^	20.44^d^	1.76^f^	6.70^gh^	4.82^e-g^
**D1 × C4**	77.95^a^	28.09^a^	14.72^fg^	1.38^a^	0.067^a^	13.64^f-h^	84.75^cd^	1.07^a^	21.64^cd^	3.73^e^	6.73^gh^	5.29^d-f^
**D2 × C1**	69.76^a^	37.18^a^	22.91^d^	0.54^a^	0.025^a^	14.18^e-g^	83.82^d^	0.91^a^	16.78^e^	3.80^e^	7.24f^g^	5.21^d-g^
**D2 × C2**	78.19^a^	29.73^a^	25.91^c^	0.68^a^	0.032^a^	11.90^f-h^	93.67^b^	1.09^a^	23.70^bc^	4.06^de^	8.26^ef^	6.04^cd^
**D2 × C3**	72.32^a^	29.92^a^	36.36^a^	0.74^a^	0.034^a^	29.13^a^	109.00^a^	1.40^a^	29.63^a^	6.62^c^	9.80^c^	6.91^bc^
**D2 × C4**	73.99^a^	28.29^a^	26.06^c^	0.88^a^	0.042^a^	19.58^b-d^	105.09^a^	1.19^a^	30.66^a^	9.05^a^	11.92^b^	7.04^b^
**D3 × C1**	65.61^a^	46.47^a^	19.53^e^	0.54^a^	0.024^a^	15.76^d-f^	84.57^cd^	0.96^a^	12.78^f^	3.79^e^	8.50^de^	5.59^de^
**D3 × C2**	69.22^a^	39.45^a^	23.50^d^	0.55^a^	0.027^da^	18.87^c-e^	91.80^bc^	1.26^a^	23.61^bc^	4.46^d^	9.59^cd^	5.79^de^
**D3 × C3**	75.12^a^	35.93^a^	33.31^b^	0.69^a^	0.030^a^	24.43^ab^	95.05^b^	1.51^a^	17.93^e^	8.02^b^	10.57^c^	7.57^b^
**D3 × C4**	68.05^a^	30.05^a^	22.02^d^	0.73^a^	0.037^a^	23.23^bc^	95.05^b^	1.49^a^	25.64^b^	7.05^c^	14.69^a^	9.83^a^

Values are means ± SD (*n* = 4). According to Duncan’s Multiple Range Test, different letters in the same column indicate significant differences among treatments (*P* ≤ 0.05). D: Drought stress, D1: 100% FC, D2: 50% FC, D3: 30% FC. C: Chitosan nanoparticles, C1: 0 ppm, C2: 30 ppm, C3: 60 ppm, C4: 90 ppm. Relative water content (RWC) and electrolyte conductivity (EC) (%), total chlorophyll (Chl), carotenoid (CAR) and soluble sugar (mg/g FW), proline and protein (μmol/g FW), total phenol and total flavonoid (FLAV) (mg/g FW), superoxide dismutase (SOD), guaiacol peroxidase (GPO), and polyphenol oxidase (PPO) (U mg^− 1^ protein)

Data presented in Fig. 1 showed that when DS was increased to 70% (30% FC), the EC of leaves increased by about 133% compared to normal irrigation conditions. At all three irrigation levels, applying different concentrations of CNPs improved EC, but this change was not statistically significant (Table 1). The lowest EC (24.19%) was measured in the untreated plants grown under regular irrigation (Fig. 1).

### Effect of DS and CNPs on photosynthetic pigments

**Fig. 2:**
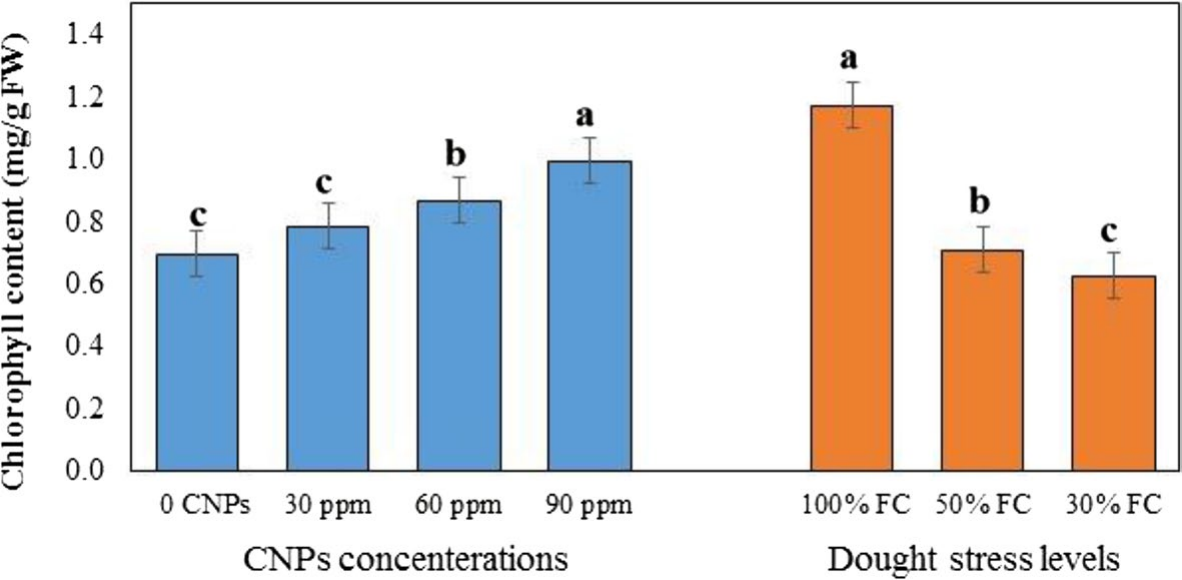
Effects of different concentration of chitosan nanoparticles (CNPs) (30, 60 and 90 ppm) and drought stress levels (30, 50, and 100% FC) on chlorophyll content in leaves of *S. abrotanoides*. Values are means ± SD (*n* = 4). According to Duncan’s Multiple Range Test, different bar letters indicate significant differences among treatments (*P* ≤ 0.05)

**Fig. 3:**
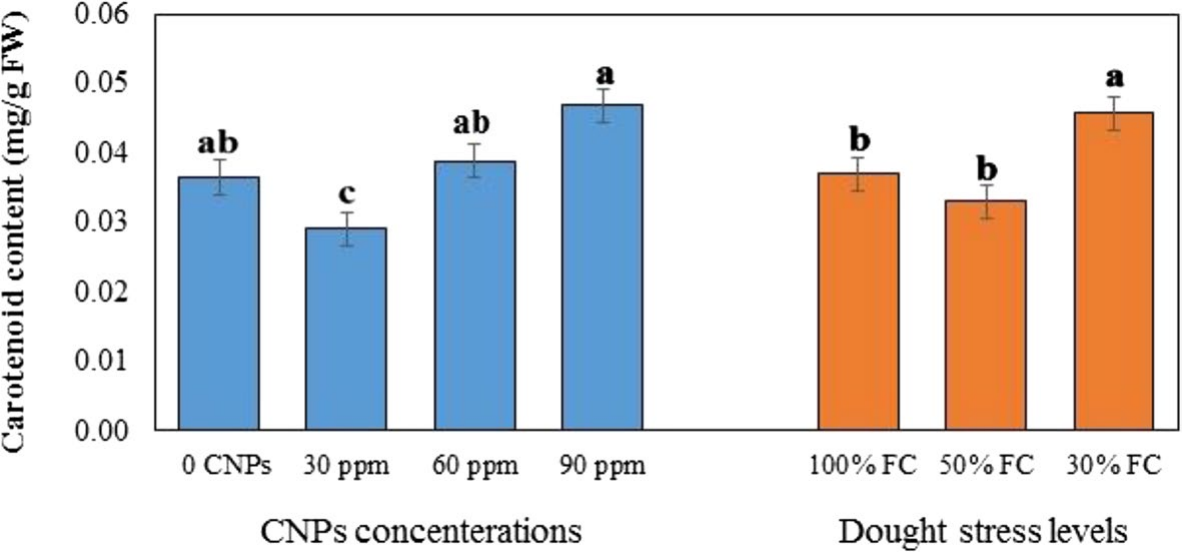
Effects of different concentration of chitosan nanoparticles (CNPs) (30, 60 and 90 ppm) and drought stress levels (30, 50, and 100% FC) on carotenoid content in leaves of *S. abrotanoides*. Values are means ± SD (*n* = 4). According to Duncan’s Multiple Range Test, different bar letters indicate significant differences among treatments (*P* ≤ 0.05)

According to the analysis of variance, irrigation stress and CNPs treatment significantly influenced photosynthetic pigments (*P* ≥ 0.05). As can be seen in Table 1. the interaction of DS and CNPs was not statistically significant. Data indicated that plants exposed to moderate and severe DS had significantly lower chlorophyll (0.54 mg/g FW) (Fig. 2) than those with regular watering, respectively. The highest carotenoid (0.046 mg/g FW) was obtained in severe DS (30% FC) (Fig. 3). In this regard, the difference between the two levels of DS (50 and 30% FC) was insignificant. In all three irrigation regimes, the chlorophyll content of the plants improved with the increase in the concentration of CNPs (Table 1., Fig. 2). However, the effect of CNPs on carotenoid content was only significant when they were applied at a concentration of 90 ppm (Table 1., Fig. 3).

### Effect of DS and CNPs on total phenol and flavonoid contents

The comparison of means showed that the plants subjected to 50 and 70% DS had significantly higher total phenol content (TPC) than the well-watered plants (control). Foliar application of CNPs in any three concentrations significantly increased TPC levels under all three irrigation regimes (Table 1). The highest TPC (109 mg GAE/g FW) was observed in the plants under 50% DS treated with 60 ppm of CNPs. This TPC level was approximately 1.3 times the TCP of the untreated plants (Table 1). Although the effect of DS, CNPs and DS × CNPs on flavonoid contents were not statistically significant (Table 1), results showed an increase in the total flavonoid content (TFC) of all of the plants subjected to DS and CNPs compared to the control.

### Effect of DS and CNPs on soluble sugar and proline content

**Fig. 4:**
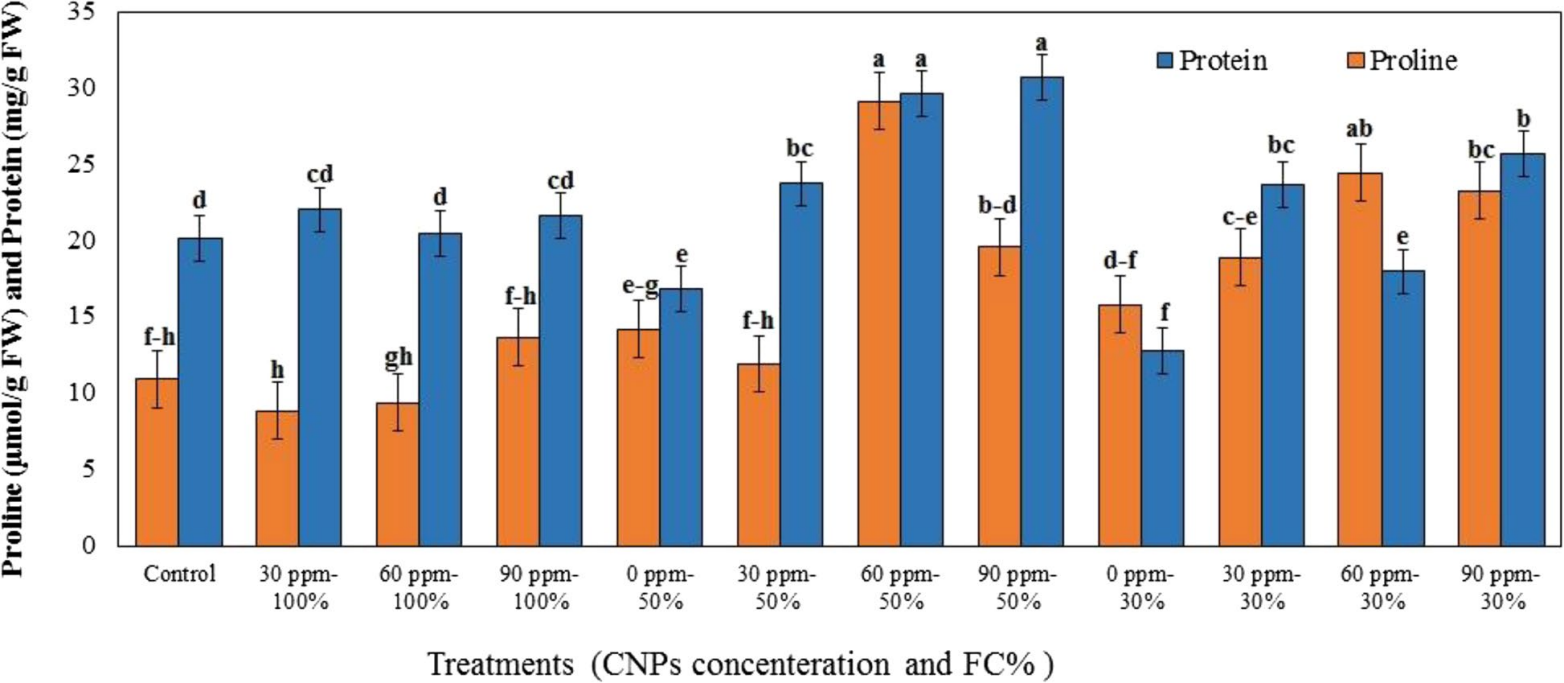
Effects of different concentration of chitosan nanoparticles (CNPs) (30, 60 and 90 ppm) on proline and protein content in leaves of *S. abrotanoides* in responses to drought stress levels (30, 50, and 100% FC). Values are means ± SD (*n* = 4). According to Duncan’s Multiple Range Test, different bar letters indicate significant differences among treatments (*P* ≤ 0.05)

The results of ANOVA showed that the interaction effect of nanoparticle concentration and irrigation regime on sugar and proline content was significant (*P* ≤ 0.05). Data presented in Table 1 showed that foliar application of CNPs at different concentrations caused a significant increase in sugar content (except for the concentration of 30 ppm under normal irrigation conditions). In all three irrigation levels, the proline content of the plants treated with higher concentrations of CNPs (60 and 90 ppm) and under DS was significantly different from that of control plants. The highest sugar content (36.36 mg/g FW) and the highest proline content (29.13 μmol/g FW) were observed in the plants treated with 60 ppm of CNPs under 50% DS. Sugar and proline contents were 1.58 and 2.05 times the sugar and proline contents of the untreated plants in the same irrigation conditions (Fig. 4).

### Effect of DS and CNPs on protein content

Results from an analysis of variance revealed that application of different concentrations of CNPs to the plants under normal irrigation conditions had no significant effect on their protein content. However, the plants subjected to 50 and 70% DS that received the CNPs had significantly higher protein content than those that did not receive the treatment (*P* ≤ 0.05). The highest protein levels were observed in the plants treated with 60 ppm and 90 ppm of CNPs under 50% DS (29.63 mg/g FW and 30.66 mg/g FW, respectively) (Table 1, Fig. 4).

### Effect of DS and CNPs on the antioxidant enzyme activity

**Fig. 5:**
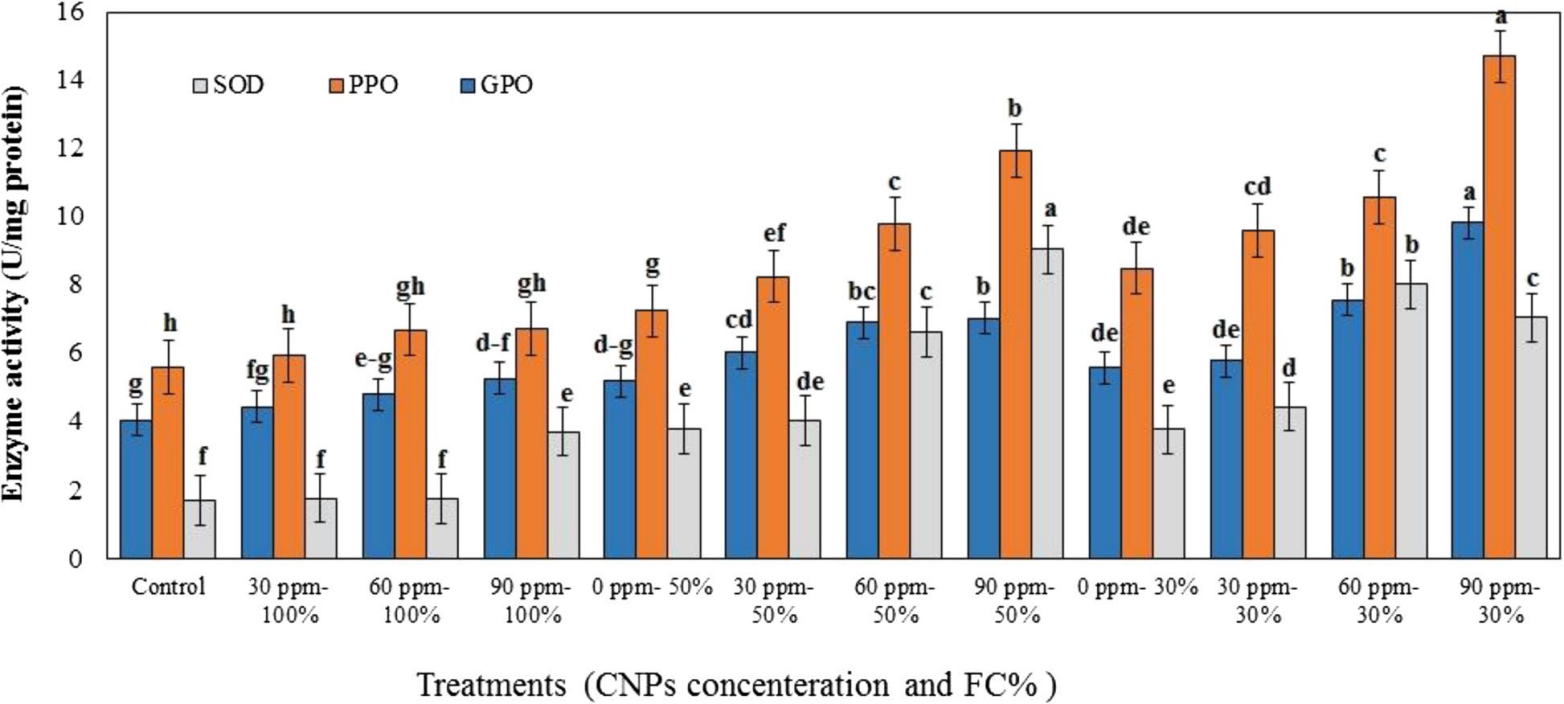
Effects of different concentration of chitosan nanoparticles (CNPs) (30, 60 and 90 ppm) on superoxide dismutase (SOD), polyphenol oxidase (PPO), and guaiacol peroxidase (GPO) activity in leaves of *S. abrotanoides* in responses to drought stress levels (30, 50, and 100% FC). Values are means ± SD (*n* = 4). According to Duncan’s Multiple Range Test, different bar letters indicate significant differences among treatments (*P* ≤ 0.05)

The plants exposed to DS showed significantly higher SOD, PPO, and GPO activity than those under normal irrigation conditions (Table 1, Fig. 5). While spraying CNPs on plants under normal watering conditions enhanced antioxidant enzyme activity, this increase was only significant for SOD and GPO when the plants were given 90 ppm of CNPs. Compared with the control plants, foliar treatment of higher doses of CNPs (60 and 90 ppm) dramatically enhanced antioxidant enzyme activity (Fig. 5). The highest SOD activity (9.05 Umg^− 1^ protein) was observed in the plants under 50% DS treated with 90 ppm of CNPs. However, data in Table 1 and Fig. 5 indicated that the highest GPO and PPO activity levels (9.83 and 14.69 Umg^− 1^ protein, respectively) were in the plants under 70% DS that received the 90 ppm treatment.

### Effect of DS and CNPs treatment on stomatal morphology

**Fig. 6:**
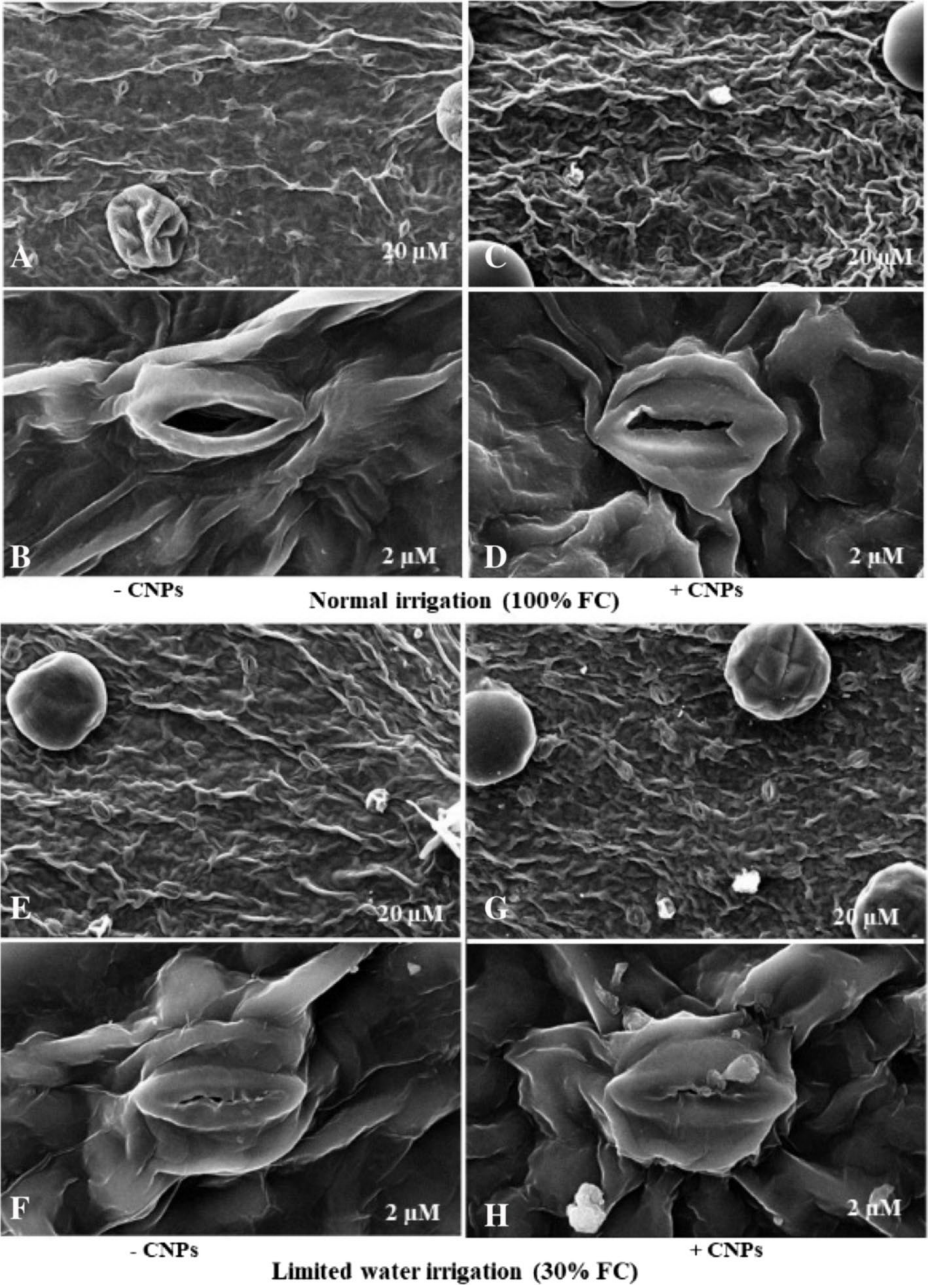
SEM images of the ultrastructure of stomata in the epidermis of *S. abrotanoides* leaves in normal (**A**, **B**) and limited water supply treatments without CNPs (**E**, **F**) or with CNPs supplementation for normal (**C**, **D**) and limited water (**G**, **H**) at 90 ppm CNPs. Chitosan nanoparticles (CNPs), Field capacity (FC). The micrographs were scaled at 2 M (**B**, **D**, **F**, and **H**) and 20 M (**A**, **C**, **E**, and **G**), respectively

Scanning electron microscopy (SEM) images of the stomatal morphology of the plants treated with 90 ppm of CNPs under two different irrigation levels (100 and 30% FC) are displayed in Fig. 6.

The results showed a notable difference in stomatal density between the leaves of the plants subjected to severe stress (40 n/mm^2^) and those of well-watered plants (37 n/mm^2^). Under normal irrigation conditions, the plants that received the CNPs treatment showed higher stomatal density (42 n/mm^2^) than untreated plants. The highest stomatal density (45 n/mm^2^) was observed in the plants treated with CNPs under DS. The plants under severe stress conditions showed a more significant stomatal aperture reduction (6 μm) than the well-watered plants (12 μm). In addition, applying the CNPs treatment under normal irrigation conditions led to a reduced stomatal aperture size (8 μm) compared to the untreated control plants. The lowest stomatal aperture size (3 μm) was measured in the plants treated with CNPs under DS. The results showed that stomatal closures were significantly higher in the water-stressed plants (19 n/mm^2^) than in the control plants (10 n/mm^2^).

The plants treated with CNPs also showed higher stomatal closures (15 n/mm^2^) than the control plants under normal irrigation conditions. The CNPs treatment reduced the number of open stomata in water-stressed plants to such an extent that the leaves of these plants had the maximum number of stomatal closures (30 n/mm^2^).

## Discussion

In this study, the effects of CNPs on DS mitigation in *S. abrotanoides* were investigated for the first time. For this purpose, the plant’s physio-biochemical and anatomical responses to various CNPs concentrations and DS levels are discussed.

Drought stress causes adverse effects on plants’ growth and biomass, RWC, membrane integrity, and photosynthetic rate [29, 30]. Different plant responses are triggered under this situation, including biochemical and physiological changes [31].

It was found low concentrations of nanomaterials (under 100 ppm) improved plant resistance to abiotic stress through mechanisms such as (1) triggering plant cell signals due to high production of ROS and/or reactive nitrogen species (RNS) and (2) stimulating the plant protection mechanism, which includes enzymatic and non-enzymatic antioxidants [32–35]. Some earlier studies have shown that CNPs at low concentrations (10–100 ppm) stimulate plant growth and defense responses to DS [14, 19, 20, 36–38]. Consequently, in this experiment, three concentrations of CNPs (30, 60, and 90 ppm) were investigated in *S. abrotanoides* under DS and compared with control plants (CNPs = 0 ppm).

However, nanoparticle application at higher concentrations has toxic effects on cellular processes and plant metabolism even under normal conditions [39–41]. For instance, CNPs at higher concentrations cause toxicity and adverse effects on plants’ growth and physiological traits [8, 17].

In our study, DS decreased the RWC of leaves (up to 20%) in *S. abrotanoides* stressed- plants. Other experiments have also reported a significant reduction in RWC under DS in *S. nemorosa* [2, 42], *S. sinaloensis* [43], and *S. miltiorrhiza* [44]. The reduced RWC of drought-stressed *S. abrotanoides* may be attributed to less access to water, decreased relative water absorption, or lower water retention in the leaves of these plants [15].

Treatment with CNPs, especially at 60 ppm, improved the RWC of the stressed plants, especially at the 70% stress level. Similarly, Behboudi et al. [15] reported that treatment of drought-stressed *Hordeum vulgare* with 90 ppm of CNPs increased RWC by 1.57 times compared to the control sample. Other investigators have also shown the positive effect of CNPs on improving RWC in *Trifolium repens* [45] and *Z. mays* [46] under DS conditions.

Chitosan nanoparticles help improves water retention and RWC of stressed plants by reducing their transpiration rate, promoting water absorption and essential elements from roots, and regulating the cell osmotic pressure. Also, CNPs benefit from stomatal conductance and photosynthetic rate, then maintain water balance in cells by stomatal adjustment [6, 47].

Contrary to the results obtained for RWC, the EC of the plants subjected to severe DS was 2.33 times higher than the plants with the regular irrigation regime. The results of this investigation revealed that plants treated with 90 ppm CNPs had a 35% lower EC than untreated plants under severe DS. These findings are consistent with the results reported by other authors [48–50]. Therefore, it is likely that CNPs reduce the lipid peroxidation and EC level of *S. abrotanoides* leaf cells suffering from DS by preserving plasma membrane structure, increasing RWC, regulating water pressure, and reducing oxidative stress [49].

The chlorophyll content is one of the most important factors in maintaining photosynthetic capacity and determining the rate of photosynthesis in plants [51, 52]. Drought stress significantly decreased total leaf chlorophyll (up to 47%) and carotenoid (up to 54.7%) content, supported by prior findings that reported comparable decreases in photosynthetic pigments of *Salvia* species under drought conditions [2, 43, 53, 54]. The reduction in chlorophyll and carotenoid levels under DS is likely due to the production of large amounts of ROS in thylakoids and the decomposition of these pigments, lipids and proteins [15, 55].

In the present study, exogenous CNPs increased chlorophyll and carotenoid concentrations during DS and had a dose-dependent effect on these photosynthesis pigments. After using 90 ppm CNPs under 50% DS, the total chlorophyll and carotenoid levels were enhanced up to 63 and 68% compared to untreated plants, respectively. Similarly, increased chlorophyll content following 90 ppm CNPs treatment under DS in *T. aestivum* has also been found by Behboudi et al. [20]. In a study by Masjedi et al. [56], CNPs increased the chlorophyll content of drought-stressed *T. aestivum* up to 33% compared with untreated plants. Ghasemi Pirbalouti et al. [18] reported that foliar application of 200 ppm of chitosan increased the carotenoid content in *Ocimum basilicum* under water limitation conditions (30% FC). There are also some reports of the positive effect of chitosan and its oligomers on the carotenoid content of *Coffea canephora* [57] and *Fragaria* × *annanasa* [58].

It appears that CNPs stimulate the expression of genes involved in the chlorophyll synthesis by increasing the internal cytokinin level and enhancing the availability of free amino compounds released from chitosan [20]. Also, chitosan foliar treatment increase ascorbate peroxidase activity, which scavenges ROS in thylakoids and reduce the breakdown of photosynthetic pigments [13, 59]. Furthermore, these nanoparticles may enhance photosynthetic efficiency by suppressing ethylene, a chlorophyllase stimulant, and increasing Rubisco carboxylase activity, which improves Rubisco activation and chlorophyll content [60].

Phenolic compounds, including flavonoids, can scavenge ROS and prevent cellular oxidation under water deficiency [21, 61]. In this experiment, DS increased the TPC and TFC of *S. abrotanoides* leaves by 29 and 13%, respectively. These results were in agreement with some scientists who founded that water deficit improved the TPC and TFC of plants, including *S. officinalis* [61], *S. sinaloensis* [43] and *S. nemorosa* [2]. However, Caser et al. [54] reported that DS reduced the level of phenolic compounds in *S. dolomitica*. Another study also stated that DS inhibited the synthesis of flavonoid compounds in the leaves and stems of *Thymus daenensis* [48]. As a result, the amount of these compounds in a stressed plant appears to be dependent on whether the plant is resistant or sensitive to DS.

This investigation showed an increase in TPC and TFC of the plants treated with CNPs in well-watered and water-stressed groups. The highest TPC (1.3-fold of control) and TFC (1.57-fold of control) were observed when 60 ppm of CNPs was applied to the plants under 50 and 70% stress levels, respectively. Similarly, a study described that the treatment of *Camellia sinensis* leaves with CNPs led to a 24% higher concentration of phenolic compounds than the untreated control group [62]. In a study by Vosoughi et al. [61], the highest TPC and TFC in drought-stressed *S. officinalis* was observed after applying 500 ppm of chitosan. There are also similar reports of the effect of chitosan and its derivatives on the concentration of phenolic compounds in other plants [63–66]. It is known that chitosan activates the PAL and TAT enzymes, important in the phenylpropanoids biosynthesis pathway and phenolic compounds against abiotic stress [21, 67, 68]. According to transcriptome data, chitosan significantly up-regulated numerous genes linked with flavonoid metabolism during DS [45].

This study indicated that soluble sugar and proline concentrations were higher in drought-stressed plants than in control. The interaction of CNPs with DS caused a significant increase in these osmoprotectants levels. At 60 ppm of CNPs and 50% DS conditions, the sugar and proline content increased 58 and 49% compared with untreated control plants, respectively. Consistent with this study, Behboudi et al. [20] demonstrated that the highest amount of proline (49.14 μmol/g FW) was observed in *T. aestivum* sprayed with 90 ppm CNPs under DS, which was 1.13 times greater than control plants. In another study, CNPs treatment led to higher proline concentration in the leaves of *C. roseus* under 50% DS [6]. In agreement with our results, Jiao et al. [69] found that using different concentrations of chitosan (50, 100 and 200 ppm) in potato plants under DS resulted in a high concentration of soluble sugar in the early phases of stress. Rabêlo et al. [21] observed a 60% rise in the sugar content in *Z. mays* plants treated with chitosan and exposed to 50% DS. There are also some reports of the interaction effect of chitosan and DS on proline and soluble sugar content in the other plants [21, 30, 48].

Under DS, the external application of chitosan and its derivatives leads to a higher accumulation of soluble sugars, proline, starch, and flavonoids in plant cells which are osmotic adjustments [3]. The higher proline amount caused by the use of CNPs might be attributed to a decrease in proline oxidation to glutamate, increase in protein turnover, or reduction in protein usage [3, 6]. Furthermore, CNPs activates proline biosynthesis genes that produce proline from glutamate [6]. Enhanced soluble sugar levels in chitosan-treated plants might be due to increased α-amylase activity, which leads to starch breakdown [59]. It has been proposed that chitosan increases the expression of many genes involved in carbohydrate transport and metabolism in *T. repens* leaves under DS [45]. As a result, chitosan might have an osmoregulatory role related to differences in sugar and energy metabolism between chitosan-treated and non-treated plants [45].

In this work, severe drought decreased total proteins by 36.5% compared to well-watered plants. There are similar reports of reduced protein content in other plants under DS [70, 71]. During DS, increased protease activity is responsible for the breakdown of proteins into amino acids, which actively includes osmotic adjustment [59]. Exogenous CNPs at 90 ppm were shown to be efficient in raising total protein to 30.66 mg/g FW (1.82-fold untreated plants) under 50% FC in the leaves of *S. abrotanoides*. This result agrees with the findings of Behboudi et al. [15, 20], who reported that using 90 ppm of CNPs improved the protein content of drought-stressed *H. vulgare* and *T. aestivum.* Li et al. [72] suggested that applying 5 and 10 ppm of CNPs increased the protein content of *T. aestivum* seedlings. In another study, 100 ppm of chitosan improved the protein content of potato plant leaves by up to 100% under DS [69] . This impact of CNPs on protein content may be related to the nitrogen content of chitosan, which plays an essential role in protein synthesis [20]. Additionally, chitosan can cause a reduction in the degradation of soluble protein by enhancing the expression of proteinase and protease inhibitor genes [69, 73].

The activity of SOD, PPO, and GPO enzymes in the current study increased in leaves of *S. abrotanoides* in response to DS*.* In accordance with our data, different plant species have observed enhancing antioxidant enzyme activity for DS-resistant [74–78]. Under DS, exogenous CNPs raised antioxidant enzyme activity in *S. abrotanoides* leaves in a dose-dependent manner (except for SOD at 60 ppm CNPs in 50% DS). Foliar application of 90 ppm of CNPs to the plants under severe DS increased the SOD, GPO, and PPO activity levels by 86, 75.8, and 72.8%, respectively (compared to untreated plants). However, the highest SOD activity was observed in the plant treated with 90 ppm of CNPs subjected to 50% FC (2.38-fold of control). Several studies support our findings that chitosan and its nanoparticles have a favorable effect on antioxidant system gene expression and activation under DS [6, 21, 34, 45, 47, 59]. In agreement with our study, Behboudi et al. [15] observed the highest SOD activity level after foliar application of 90 ppm of CNPs to drought-stressed barley plants. Chandra et al. [62] found that 24 hours after treating *Camellia. sinensis* leaves with 10 ppm CNPs, POX, SOD and PPO activity levels of these plants were 4, 1.7 and 3.5 times than untreated control plants. In the same way, Abdel-Aziz [79] reported that using CNPs significantly increased the activity of antioxidant enzymes, including peroxidase and PPO in *Vicia faba*. Similar results showed that chitosan increases the activity of antioxidant enzymes, including SOD, POX and GPO, in other plants [21, 49, 69, 80]. So, it has been suggested that CNPs are potent inducers of antioxidative enzymes activity under abiotic stress [9] . However, Ali et al. [81] discovered that PPO activity reduced in *Rosa damascena* Mill. flowers treated with CNPs, suggesting that CNPs may play a role in floral quality maintenance.

The positively charged chitosan binds to a negatively charged phospholipid or protein receptor in the cell membrane, inducing oxidative burst and cascade reactions. ROS act as signaling molecules, which activate the antioxidant enzyme systems [21, 29]. CNPs reduce ROS accumulation and its destructive effects under DS by acting on nucleus and chloroplast genes, modifying the chromatin and resulting in higher photosynthetic and enzymatic antioxidant activity [14, 15].

In this examination, the plants subjected to severe DS showed 1.08-fold higher stomatal density and 2-fold smaller stomatal aperture size than well-watered plants. There are similar reports of reduced aperture size due to DS in other plants [82, 83]. Similarly, previous works found an increase in stomatal density after applying DS to some plants [83] . Our results were opposed to some studies that have reported that DS reduces the stomatal density of leaves [82, 84]. Although environmental factors and growth stages strongly influence stomatal density and aperture size, plant genotype also impacts these factors [83, 85]. Results of this investigation showed that foliar application of CNPs reduced stomatal aperture size by 33 and 50% in plants under normal and water stress conditions, respectively, compared with untreated control plants. Pitoyo et al. [86] also observed a similar decrease in the stomatal aperture size in *Grammatophyllum scriptum* after applying chitosan. Our finding is also consistent with Lee et al. [87] results, which reported that the foliar application of chitosan reduced the stomatal aperture size in tomato leaves by up to 62%. Elicitors like chitosan stimulate ROS production and especially H_2_O_2_ in stomatal guard cells, making the plant respond by reducing the stomatal aperture size [87]. However, this conclusion does not agree with the findings of a study on the effect of CNPs on stomatal aperture size in *Coffea canephora*, which stated that CNPs might increase the stomatal aperture size [38].

Our findings revealed that foliar application of CNPs to *S. abrotanoides* increased stomatal density about 12% in both water regimes, compared to untreated plants. Similarly, a study by Hasanah and Sembiring [88] reported that chitosan increased stomatal density in various soybean cultivars. It is possible that by increasing stomatal density and reducing stomatal aperture size, chitosan facilitates the rapid opening and closure of stomata, thus increasing stomatal conductance, reducing water evaporation, and providing the CO_2_ required for photosynthesis in DS conditions [83].

This survey found that, the number of stomatal closures in stressed plants was as much as 57% higher than the corresponding number in the control plants, consistent with the results reported for other plants [54, 82]. Stomatal closure is one of the most effective mechanisms for reducing water loss through transpiration under water stress conditions. It also reduces photosynthesis and CO_2_ absorption. However, having a greater number of closed stomata and lower aperture size decreases the plant’s water requirement and makes it more tolerant to water stress [54].

This research indicated that CNPs increased the number of stomatal closures under both regular irrigation and severe drought conditions. The highest number of stomatal closures (30 n/mm^2^) was observed in the CNPs treated plant subjected to drought conditions. Likewise, foliar treatment of chitosan nanoemulsion on water-stressed pearl millet plants induced more stomata to close down than untreated plants [10]. According to the earlier study, foliar application of chitosan in *Capsicum annuum* resulted in less open stomata than plants not treated with this elicitor in DS conditions [89].

Chitosan can efficiently contribute to stomata closure and decrease transpiration, preserving leaf water under drought conditions by increasing ROS and reactive nitrogen species (RNS) in stomatal guard cells and up-regulating ABA synthesis [10, 11]. Furthermore, chitosan facilitates the stomatal closure process by inhibiting the synthesis of WRKY17 transcription factors under water stress conditions [90].

## Conclusion

For the first time, this study revealed information related to the *S. abrotanoides* tolerance mechanisms induced by CNPs under moderate and severe DS. CNPs (particularly at 60 and 90 ppm) improved drought resistance in *S. abrotanoides*, as evidenced by considerable increases in biochemical and physiological responses under DS. Due to the environmental benefits of CNPs (e.g., natural origin, nontoxicity, safety, and biodegradability), they can be an excellent alternative for agrochemicals to mitigate DS. Future investigations should evaluate the molecular mechanisms and expression pattern of related genes induced in this ornamental-medicinal plant after using CNPs for water deficit tolerance.

## Methods

### Nanoparticles characterization

**Fig. 7:**
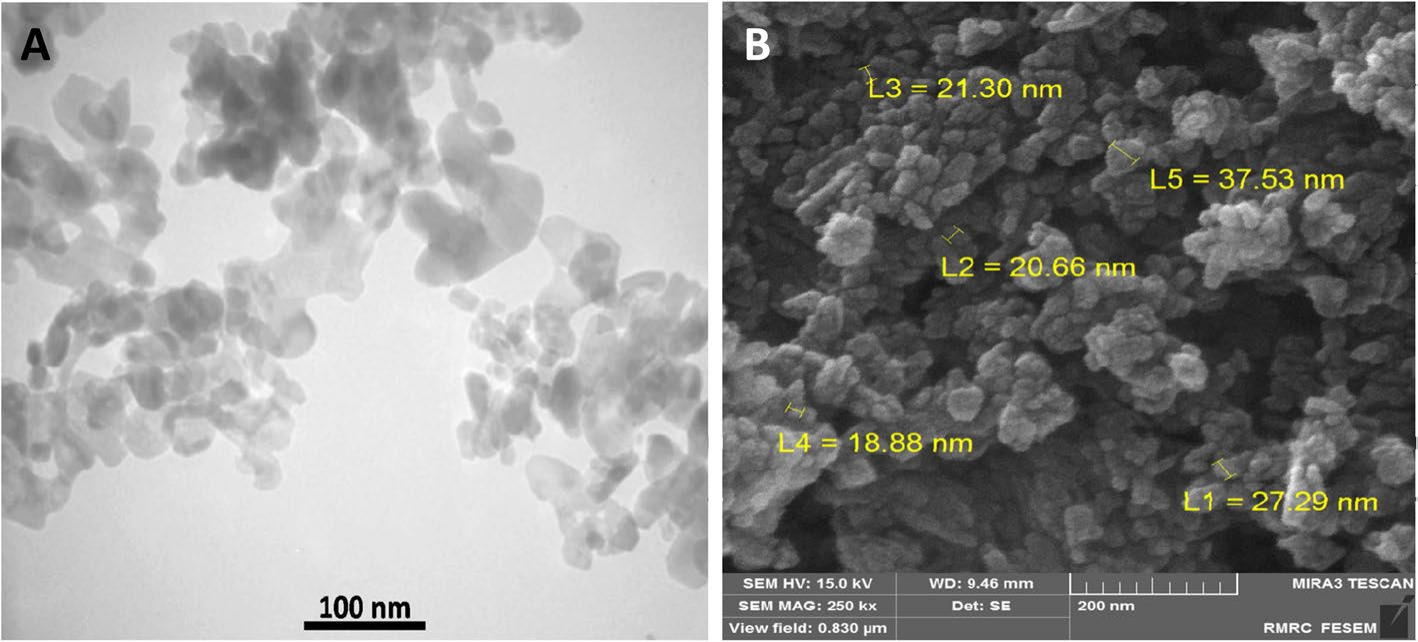
**A**: TEM image of chitosan nanoparticles; **B**: SEM image of chitosan nanoparticles

Chitosan nanoparticles were commercially purchased from Iranian Nanomaterials Pioneers Company, NANOSANY (Mashhad, Iran). The characteristics of CNPs were 50 nm in size, with a molecular weight of 161 g/mol and 99% purity (Supplementary files 2, 3, 4, 5, 6 and 7). Figure 7 represents the Transmission Electron Microscope (TEM) (A) and Scanning Electron Microscope (SEM) (B) images of CNPs, respectively. The CNPs were synthesized by NANOSANY Company as described by Ghade et al. [91] (Supplementary Fig. 1, 2, 3, 4, 5 and 6).

### Preparation of nanoparticle suspension

To prepare a suspension of CNPs, the nanoparticles were dissolved in 1% acetic acid, then diluted with distilled water, and heated for 2 hours at 90 °C at 1200 rpm. The pH of the solution was adjusted with 1 N sodium hydroxide (pH = 6.5–7) [92].

### Plant materials, cultivation conditions, and treatments

Seeds of mature *S. abrotanoides* were collected from its natural habitat in Kalat Naderi County (Khorasan Razavi province, Iran) at the longitude of 59° 9′ 40“ E, latitude of 36° 24’ 20” N, and altitude of 2100 m above sea level. Seeds were grown in growing trays containing cocopeat (80%) and perlite (20%). The four-leaf stage seedlings were transferred to plastic pots, filled with sand and clay loam soil (1:1; v/v, pH = 7.65, and EC = 1.55 dSm^− 1^). Plants was fertilized after seeding with a NPK fertilizer (20–20-20, 2 g/l). The average temperature of the greenhouse was 18–24 °C (night-day), the photoperiod was 14–16 h, and the humidity was 50–60%.

**Fig. 8:**
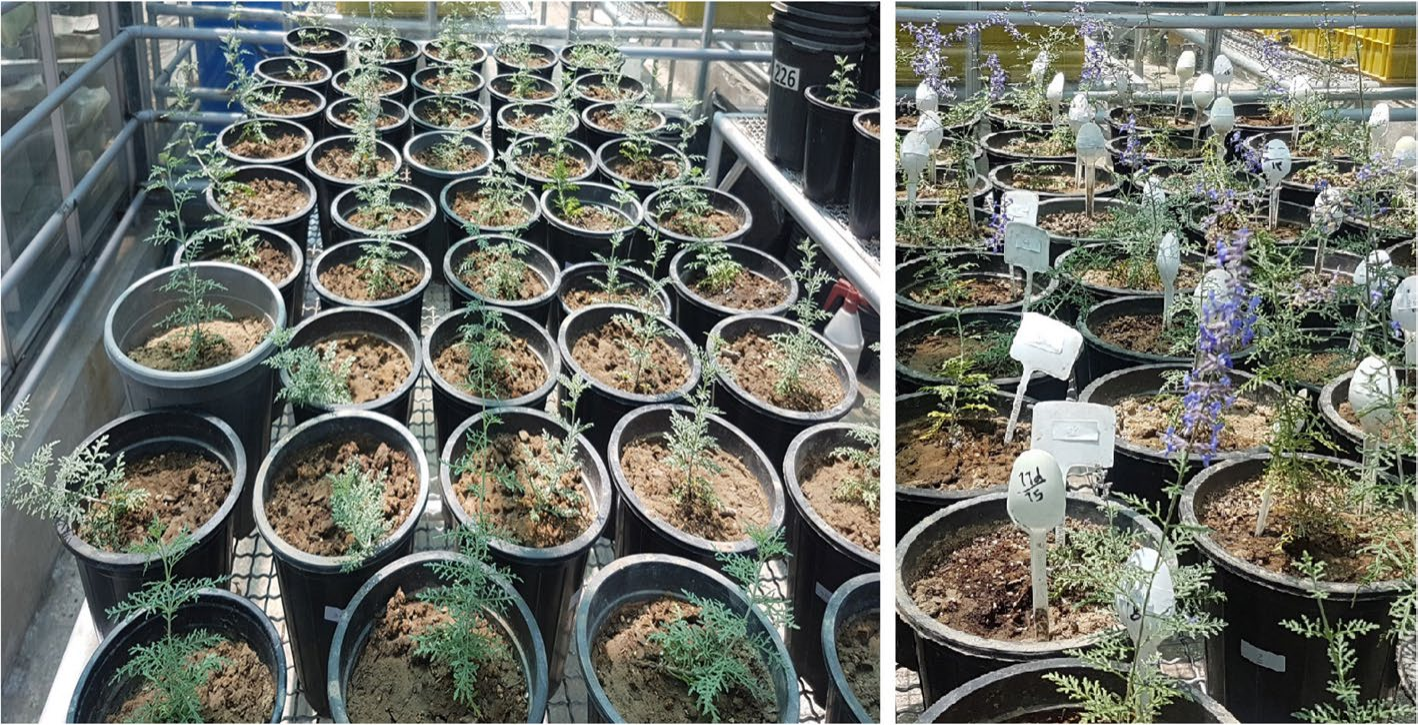
Treated plants (*Salvia abrotanoides*) with different concentration of chitosan nanoparticles (30, 60 and 90 ppm) exposed to (100, 50 and 30% FC)

Chitosan nanoparticles were prepared at four concentrations (0, 30, 60 and 90 ppm) [15, 20, 37, 38], and their effect in three irrigation conditions: 30% of the field capacity (FC) (severe DS), 50% FC (medium DS), and 100% FC (well-watered) were investigated. All experiments were conducted in the greenhouse of the Plant Science Research Center of Ferdowsi University of Mashhad, Iran in 2020. Soil moisture content was measured according to gravimetric method [93] and monitored daily until the end of experiment using soil moisture meter (EXTECH MO750, USA, probe length: 20 cm probe and max resolution: 0.1%). The pots were irrigated by tap water (when the humidity dropped below a certain level) to keep the moisture content at the desired level (100, 50 and 30% FC). Upon the appearances of first flower buds, the plants were sprayed with different concentrations of CNPs (Fig. 8). DS was applied to the plants 1 week after the first CNPs treatment and the second foliar application of CNPs was carried out on the first day after the plants exposed to water stress. The plants were collected 1 month after the start of DS. The leaves of the collected plants were immediately frozen in liquid nitrogen and then stored in a freezer at − 80 °C for phytochemical experiments.

### Structural analysis of leaves with scanning electron microscopy (SEM)

At the end of the experiment (1 month following the initiation of DS at full flowering stage), a SEM (LEO 1450VP, Germany) was used to examine the leaf structure and the number and function of stomata. The samples used for this purpose were stabilized with glutaraldehyde [94].

### Determination of chlorophyll and carotenoid contents

The chlorophyll content of the samples was measured using the method of Lichtenthaler [95]. For this purpose, 1 ml of acetone (80%) was added to 0.025 g of fresh leaf samples and the mix was thoroughly ground and homogenized. The product was centrifuged at 3000 g for 5 min, and the absorbance of the supernatant was read at 470, 645 and 663 nm using a spectrophotometer (UV-VIS, Optima SP-3000 Plus, Bratislava, Slovakia). The content of chlorophyll and carotenoids was measured in mg per gram of fresh weight (mg/g FW).

### Measurement of relative water content (RWC) and electrolyte conductivity (EC)

To measure the RWC of leaves as instructed by Ritchie et al. [96], fully mature leaves were cut from the plants and immediately weighed to determine their fresh weight (FW). The leaves were then placed in distilled water at 4 °C for 24 h and weighed again to determine their turgid weight (TW). The final weighing was done after placing the leaves in an oven at 70 °C for 48 h to determine the dry weight (DW). These measurements were then used to calculate RWC by the following formula:

$$\mathrm{RWC}=\left[\left(\mathrm{FW}-\mathrm{DW}\right)/\left(\mathrm{TW}-\mathrm{DW}\right)\right]\times 100$$

One gram of leaf tissue was added to 20 ml of distilled water and maintained at room temperature (24 °C) for 24 h to determine the EC of leaves. After 24 h, the electrical conductivity of the mixture was measured by an EC meter to determine the initial EC (EC1). The Secondary EC (EC2) was determined by repeating the measurement after placing the sample in an autoclave (120 °C) for 15 min. With this data, the electrolyte conductivity of the leaves was calculated using the formula below [97].

$$\mathrm{EC}=\left(\mathrm{EC}1/\mathrm{EC}2\right)\times 100$$

### Determination of chlorophyll and carotenoid contents

The chlorophyll content of the samples was measured using the method of Lichtenthaler [95]. For this purpose, 1 ml of acetone (80%) was added to 0.025 g of fresh leaf samples and the mix was thoroughly ground and homogenized. The product was centrifuged at 3000 g for 5 min, and the absorbance of the supernatant was read at 470, 645 and 663 nm using a spectrophotometer (UV-VIS, Optima SP-3000 Plus, Bratislava, Slovakia). The content of chlorophyll and carotenoids was measured in mg/g FW.

### Determination of total phenol and flavonoid contents

Fresh leaves (0.1 g) were thoroughly homogenized in 1 ml methanol and stored at 4 °C for 24 h to create the sample extract. The supernatant was removed for use in phenol and flavonoid assays after centrifugation at 6000 rpm for 15 min. The total phenol content (TPC) was determined using the method of Wojdyło et al. [98] with a slight modification. For this purpose, 100 μL of the extract (100 mg/ml) was mixed with 200 μL of the Folin-Ciocâlteu reagent (50%) and 100 μL of distilled water. After 3 min, 1 ml of 20% sodium carbonate was added and the mixture was placed in the dark for 1 h. The absorbance of the samples was then read at 765 nm with a spectrophotometer against a blank sample (without the extract). Finally, TPC of the samples was determined based on the gallic acid standard curve in equivalent mg/g FW (Supplementary File 1). To determine the total flavonoid content (TFC), 100 μl of the extract was mixed with 300 μl of methanol, then with 20 μl of aluminum chloride (10%) and 20 μl of potassium acetate (1 M), and the mixture was brought to a volume of 1 ml with distilled water. After 30 min, the absorbance of the samples at 517 nm was read with a spectrophotometer. TFC of the extract was calculated based on the standard quercetin curve in equivalent mg/g FW [99] (Supplementary File 1).

### Measurement of soluble sugar

For this measurement, 0.01 g of fresh leaves were homogenized with 1 ml of ethanol (70%) and kept at 4 °C for 1 week. The extract was centrifuged at 6000 rpm for 15 min and the supernatant was separated for measuring the concentration of soluble sugar. This measurement was performed using the phenol-sulfuric acid method. This method is based on the acid hydrolysis of soluble sugars and the formation of furfural, which produces a color complex with phenol. For this purpose, 100 ml of the extract was combined with 300 ml of distilled water and 1 ml of phenol (5%) and vortexed before adding 1 ml of concentrated sulfuric acid. After 30 min, the absorbance of the sample at 485 nm was read against a blank sample. The sugar contents were determined using a standard curve in mg/g FW. To determine the concentration of soluble sugar, first, a standard curve with different glucose concentrations was prepared (Supplementary File 1). It was then used to determine the unknown concentrations in the studied solutions [100].

### Determination of proline content

Proline content was determined using the method of Bates et al. [101]. For this purpose, 0.1 g of fresh leaf tissue was ground on ice with 1 ml of sulfosalicylic acid (3%) to homogenize. The resulting product was centrifuged at 3000 g and 4 °C for 5 min. After separating the supernatant, 200 μl of it was mixed with 200 μl of ninhydrin reagent and 200 μl of glacial acetic acid. The mixture was placed in a 100 °C water bath for 30 min and then immediately cooled on ice. Once cooled, the product was mixed with 600 μl of toluene and vortexed for 30 sec. This caused the contents of the tube to split into two phases (opaque proline-containing toluene phase at the top and clear phase at the bottom). After 20 min, the optical absorbance of the upper phase was read at 520 nm and the concentration of proline in the solution was determined using the standard proline curve (Supplementary File 1). Finally, proline content in micromoles per gram of fresh plant weight (μmol/g FW) was calculated using the following formula [101].

$$\mathrm{Proline}\ \mathrm{Content}\ \left(\mathrm{in}\ \mathrm{micromoles}\ \mathrm{per}\ \mathrm{gram}\ \mathrm{of}\ \mathrm{fresh}\ \mathrm{plant}\ \mathrm{weight}\right)=\left(\frac{\upmu \mathrm{g}\ \mathrm{prolin}}{\mathrm{ml}}\times \frac{\mathrm{ml}\ \mathrm{toloen}}{115.5\ \left(\upmu \mathrm{g}/\upmu \mathrm{mol}\right)}\right)/\frac{\mathrm{gr}\ \mathrm{sample}}{5}\Big)$$

### Preparation of enzyme extract and measurement of total protein

Fresh leaves (0.05 g) were weighed and completely powdered with liquid nitrogen to make the enzyme extract. Then 1 ml of the phosphate-buffered saline (PBS) (50 mM, pH = 7.8) and ethylene diamine tetra acetic acid (EDTA) with a concentration of 0.1 mM were added, and the mixture was thoroughly homogenized. The product was centrifuged at 12000 g and 4 °C for 15 min. The supernatant was separated and stored in a freezer at − 80 °C to measure protein and enzyme activity. To determine the amount of protein in the extract, 2.5 ml of Bradford solution was added to 50 μl of the separated supernatant, and the absorbance was read at 595 nm after 5 min to determine the amount of protein in the extract. Specific concentrations of standard bovine serum albumin (BSA) were prepared in the extraction buffer to create a protein calibration curve [102, 103] (Supplementary File 1).

### Assay of antioxidant enzyme activity

Superoxide dismutase (SOD) activity was assayed by measuring the inhibition of photochemical reduction of nitroblutetrazolium (NBT) in the reaction mixture according to Giannopolitis and Ries [104] with slight modifications. For this measurement, the reaction mixture comprised phosphate buffer (pH = 7.8, 50 mM), methionine (13 mM), nitrobltetrazolium (75 mM), riboflavin (2 μM), sodium carbonate (50 mM), triton X-100 (0.025%), and 50 μL of the enzyme extract. The mixture was exposed to light (20 W) for 15 min and then absorbance was measured at 560 nm against the blank sample (not exposed to light). The production of aqueous formazan was measured by measuring the increase in absorbance and the result was expressed in terms of enzymatic unit per mg of protein (U mg^− 1^ protein).

Polyphenol oxidase (PPO) activity was measured using the method of Raymond et al. [105] with some modifications. For this measurement, the reaction mixture consisted of phosphate buffer (pH = 7, 50 mM), pyrogallol (0.02 M), and 50 μL of the enzyme extract. The amount of purpurogallin formed was determined by measuring absorbance at 420 nm every 5 sec for 1 min and the result was expressed in terms of U mg^− 1^ protein.

The guaiacol peroxidase (GPO) activity was measured by the method of Hemeda and Klein [106] with some modifications. In this measurement, the reaction mixture comprised phosphate buffer (pH = 6, 20 mM), guaiacol (5 mM), H_2_O_2_ (1 mM), and 50 μL of the enzyme extract. Enzyme activity was determined by measuring the increase in absorbance at 470 nm due to guaiacol oxidation. The change in absorption was measured every 5 sec for 1 min and the result was expressed in U mg^− 1^ protein.

### Statistical analysis

The research was conducted according to a factorial experiment (4 × 3) based on completely randomized design with four replications. Factors including nanoparticle concentration (0, 30, 60 and 90 ppm) and DS (30, 50 and 100% FC). For data analysis, the analysis of variance (ANOVA) was performed using the software SPSS version 21, and the comparison of means for the measured traits was performed using the Duncan’s test at test at a significance level of 5%. The graphs were drawn with the software Excel.

## Supplementary Information

**Additional file 1.** Report of analysis of variance and mean comparisons using Minitab.

**Additional file 2. **Some photos of *Salvia abrotanoides* in the green house and field in Iran.

**Additional file 3: Figure 1**. Standard curve of gallic acid. **Figure 2**. Standard curve of quercetin. **Figure 3**. Standard curve of glucose. **Figure 4**. Standard curve of proline. **Figure 5**. Standard curve of bovine serum albumin (BSA).

**Additional file 4.**

**Additional file 5.**

**Additional file 6.**

**Additional file 7.**

**Additional file 8.**

**Additional file 9.**

**Additional file 10.**

**Additional file 11.**

**Additional file 12.**

**Additional file 13.**

**Additional file 14.**

**Additional file 15.**

## Data Availability

All of the data supporting our research findings are contained in the methods section of the manuscript. Details are provided in the attached additional files.

## Abbreviations

DS: Drought stress
CNPs: Chitosan nanoparticles
FC: Field capacity
EC: Electrolyte Conductivity
RWC: Relative Water Content
TPC: Total phenol content
TFC: Total flavonoid content
SOD: Superoxide dismutase
PPO: Polyphenol oxidase
GPO: Guaiacol peroxidase
SEM: Scanning Electron Microscopy
ROS: Reactive oxygen species
