# Supplementary material for: Chitosan nanoparticles improve physiological and biochemical responses of Salvia abrotanoides (Kar.) under drought stress
Source: BMC Plant Biol. 2022 Jul 22;22:364. doi: 10.1186/s12870-022-03689-4 (PMC9308334; doi:10.1186/s12870-022-03689-4)
Supplement: Supplementary file 1 — Additional file 1. Report of analysis of variance and mean comparisons using Minitab. [file 12870_2022_3689_MOESM1_ESM.doc]

**Report of analysis of variance and mean comparisons using Minitab**

**One-way ANOVA: pH versus TRT**

Method

| Null hypothesis | All means are equal |
| --- | --- |
| Alternative hypothesis | Not all means are equal |
| Significance level | α = 0.05 |

*Equal variances were assumed for the analysis.*

Factor Information

| Factor | Levels | Values |
| --- | --- | --- |
| TRT | 9 | 1; 2; 3; 4; 5; 6; 7; 8; 9 |

Analysis of Variance

| Source | DF | Adj SS | Adj MS | F-Value | P-Value |
| --- | --- | --- | --- | --- | --- |
| TRT | 8 | 1.8109 | 0.22636 | 3.24 | 0.050 |
| Error | 9 | 0.6291 | 0.06990 |  |  |
| Total | 17 | 2.4400 |  |  |  |

Model Summary

| S | R-sq | R-sq(adj) | R-sq(pred) |
| --- | --- | --- | --- |
| 0.264378 | 74.22% | 51.30% | 0.00% |

Means

| TRT | N | Mean | StDev | 95% CI |
| --- | --- | --- | --- | --- |
| 1 | 2 | 5.851 | 0.545 | (5.429; 6.274) |
| 2 | 2 | 6.8165 | 0.0219 | (6.3936; 7.2394) |
| 3 | 2 | 6.838 | 0.305 | (6.415; 7.260) |
| 4 | 2 | 6.9055 | 0.1294 | (6.4826; 7.3284) |
| 5 | 2 | 6.510 | 0.385 | (6.087; 6.932) |
| 6 | 2 | 6.608 | 0.228 | (6.186; 7.031) |
| 7 | 2 | 6.84050 | 0.00495 | (6.41760; 7.26340) |
| 8 | 2 | 6.8860 | 0.1004 | (6.4631; 7.3089) |
| 9 | 2 | 6.8300 | 0.1047 | (6.4071; 7.2529) |

*Pooled StDev = 0.264378*

**Fisher Pairwise Comparisons**

Grouping Information Using the Fisher LSD Method and 95% Confidence

| TRT | N | Mean | Grouping | |
| --- | --- | --- | --- | --- |
| 4 | 2 | 6.9055 | A |  |
| 8 | 2 | 6.8860 | A |  |
| 7 | 2 | 6.84050 | A |  |
| 3 | 2 | 6.838 | A |  |
| 9 | 2 | 6.8300 | A |  |
| 2 | 2 | 6.8165 | A |  |
| 6 | 2 | 6.608 | A |  |
| 5 | 2 | 6.510 | A |  |
| 1 | 2 | 5.851 |  | B |

*Means that do not share a letter are significantly different.*

**One-way ANOVA: RWC versus TRT**

Method

| Null hypothesis | All means are equal |
| --- | --- |
| Alternative hypothesis | Not all means are equal |
| Significance level | α = 0.05 |

*Equal variances were assumed for the analysis.*

Factor Information

| Factor | Levels | Values |
| --- | --- | --- |
| TRT | 9 | 1; 2; 3; 4; 5; 6; 7; 8; 9 |

Analysis of Variance

| Source | DF | Adj SS | Adj MS | F-Value | P-Value |
| --- | --- | --- | --- | --- | --- |
| TRT | 8 | 4805.0 | 600.62 | 13.17 | 0.000 |
| Error | 9 | 410.5 | 45.61 |  |  |
| Total | 17 | 5215.4 |  |  |  |

Model Summary

| S | R-sq | R-sq(adj) | R-sq(pred) |
| --- | --- | --- | --- |
| 6.75338 | 92.13% | 85.13% | 68.52% |

Means

| TRT | N | Mean | StDev | 95% CI |
| --- | --- | --- | --- | --- |
| 1 | 2 | 83.58 | 4.13 | (72.78; 94.38) |
| 2 | 2 | 56.70 | 6.58 | (45.89; 67.50) |
| 3 | 2 | 77.62 | 10.73 | (66.81; 88.42) |
| 4 | 2 | 55.36 | 5.71 | (44.55; 66.16) |
| 5 | 2 | 79.73 | 5.01 | (68.93; 90.53) |
| 6 | 2 | 81.9400 | 0.0424 | (71.1374; 92.7426) |
| 7 | 2 | 46.74 | 1.99 | (35.94; 57.54) |
| 8 | 2 | 38.965 | 0.898 | (28.162; 49.768) |
| 9 | 2 | 49.31 | 13.14 | (38.51; 60.11) |

*Pooled StDev = 6.75338*

**Fisher Pairwise Comparisons**

Grouping Information Using the Fisher LSD Method and 95% Confidence

| TRT | N | Mean | Grouping | | |
| --- | --- | --- | --- | --- | --- |
| 1 | 2 | 83.58 | A |  |  |
| 6 | 2 | 81.9400 | A |  |  |
| 5 | 2 | 79.73 | A |  |  |
| 3 | 2 | 77.62 | A |  |  |
| 2 | 2 | 56.70 |  | B |  |
| 4 | 2 | 55.36 |  | B |  |
| 9 | 2 | 49.31 |  | B | C |
| 7 | 2 | 46.74 |  | B | C |
| 8 | 2 | 38.965 |  |  | C |

*Means that do not share a letter are significantly different.*

**One-way ANOVA: CHLOROPHYL versus TRT**

Method

| Null hypothesis | All means are equal |
| --- | --- |
| Alternative hypothesis | Not all means are equal |
| Significance level | α = 0.05 |

*Equal variances were assumed for the analysis.*

Factor Information

| Factor | Levels | Values |
| --- | --- | --- |
| TRT | 9 | 1; 2; 3; 4; 5; 6; 7; 8; 9 |

Analysis of Variance

| Source | DF | Adj SS | Adj MS | F-Value | P-Value |
| --- | --- | --- | --- | --- | --- |
| TRT | 8 | 4.7882 | 0.59853 | 10.95 | 0.001 |
| Error | 9 | 0.4919 | 0.05466 |  |  |
| Total | 17 | 5.2801 |  |  |  |

Model Summary

| S | R-sq | R-sq(adj) | R-sq(pred) |
| --- | --- | --- | --- |
| 0.233789 | 90.68% | 82.40% | 62.73% |

Means

| TRT | N | Mean | StDev | 95% CI |
| --- | --- | --- | --- | --- |
| 1 | 2 | 0.27750 | 0.01344 | (-0.09647; 0.65147) |
| 2 | 2 | 0.1065 | 0.0304 | (-0.2675; 0.4805) |
| 3 | 2 | 1.3770 | 0.0764 | (1.0030; 1.7510) |
| 4 | 2 | 1.4365 | 0.0332 | (1.0625; 1.8105) |
| 5 | 2 | 1.2735 | 0.0898 | (0.8995; 1.6475) |
| 6 | 2 | 0.49100 | 0.00990 | (0.11703; 0.86497) |
| 7 | 2 | 0.1415 | 0.0573 | (-0.2325; 0.5155) |
| 8 | 2 | 1.17950 | 0.00636 | (0.80553; 1.55347) |
| 9 | 2 | 0.700 | 0.687 | (0.326; 1.074) |

*Pooled StDev = 0.233789*

**Fisher Pairwise Comparisons**

Grouping Information Using the Fisher LSD Method and 95% Confidence

| TRT | N | Mean | Grouping | | | |
| --- | --- | --- | --- | --- | --- | --- |
| 4 | 2 | 1.4365 | A |  |  |  |
| 3 | 2 | 1.3770 | A |  |  |  |
| 5 | 2 | 1.2735 | A |  |  |  |
| 8 | 2 | 1.17950 | A | B |  |  |
| 9 | 2 | 0.700 |  | B | C |  |
| 6 | 2 | 0.49100 |  |  | C | D |
| 1 | 2 | 0.27750 |  |  | C | D |
| 7 | 2 | 0.1415 |  |  |  | D |
| 2 | 2 | 0.1065 |  |  |  | D |

*Means that do not share a letter are significantly different.*

**One-way ANOVA: ASCORBIC ACID versus TRT**

Method

| Null hypothesis | All means are equal |
| --- | --- |
| Alternative hypothesis | Not all means are equal |
| Significance level | α = 0.05 |

*Equal variances were assumed for the analysis.*

Factor Information

| Factor | Levels | Values |
| --- | --- | --- |
| TRT | 9 | 1; 2; 3; 4; 5; 6; 7; 8; 9 |

Analysis of Variance

| Source | DF | Adj SS | Adj MS | F-Value | P-Value |
| --- | --- | --- | --- | --- | --- |
| TRT | 8 | 65.46 | 8.182 | 3.49 | 0.040 |
| Error | 9 | 21.12 | 2.346 |  |  |
| Total | 17 | 86.57 |  |  |  |

Model Summary

| S | R-sq | R-sq(adj) | R-sq(pred) |
| --- | --- | --- | --- |
| 1.53177 | 75.61% | 53.93% | 2.43% |

Means

| TRT | N | Mean | StDev | 95% CI |
| --- | --- | --- | --- | --- |
| 1 | 2 | 1.000 | 0.173 | (-1.450; 3.451) |
| 2 | 2 | 4.690 | 0.218 | (2.240; 7.140) |
| 3 | 2 | 1.338 | 0.697 | (-1.112; 3.788) |
| 4 | 2 | 6.23 | 3.74 | (3.78; 8.68) |
| 5 | 2 | 2.005 | 0.992 | (-0.446; 4.455) |
| 6 | 2 | 5.768 | 0.668 | (3.317; 8.218) |
| 7 | 2 | 3.931 | 0.404 | (1.481; 6.382) |
| 8 | 2 | 2.376 | 0.215 | (-0.074; 4.826) |
| 9 | 2 | 5.58 | 2.22 | (3.13; 8.03) |

*Pooled StDev = 1.53177*

**Fisher Pairwise Comparisons**

Grouping Information Using the Fisher LSD Method and 95% Confidence

| TRT | N | Mean | Grouping | | | |
| --- | --- | --- | --- | --- | --- | --- |
| 4 | 2 | 6.23 | A |  |  |  |
| 6 | 2 | 5.768 | A | B |  |  |
| 9 | 2 | 5.58 | A | B |  |  |
| 2 | 2 | 4.690 | A | B | C |  |
| 7 | 2 | 3.931 | A | B | C | D |
| 8 | 2 | 2.376 |  | B | C | D |
| 5 | 2 | 2.005 |  |  | C | D |
| 3 | 2 | 1.338 |  |  | C | D |
| 1 | 2 | 1.000 |  |  |  | D |

*Means that do not share a letter are significantly different.*

**One-way ANOVA: APTI versus TRT**

Method

| Null hypothesis | All means are equal |
| --- | --- |
| Alternative hypothesis | Not all means are equal |
| Significance level | α = 0.05 |

*Equal variances were assumed for the analysis.*

Factor Information

| Factor | Levels | Values |
| --- | --- | --- |
| TRT | 9 | 1; 2; 3; 4; 5; 6; 7; 8; 9 |

Analysis of Variance

| Source | DF | Adj SS | Adj MS | F-Value | P-Value |
| --- | --- | --- | --- | --- | --- |
| TRT | 8 | 53.44 | 6.680 | 5.22 | 0.012 |
| Error | 9 | 11.51 | 1.279 |  |  |
| Total | 17 | 64.95 |  |  |  |

Model Summary

| S | R-sq | R-sq(adj) | R-sq(pred) |
| --- | --- | --- | --- |
| 1.13098 | 82.28% | 66.52% | 29.10% |

Means

| TRT | N | Mean | StDev | 95% CI |
| --- | --- | --- | --- | --- |
| 1 | 2 | 8.976 | 0.254 | (7.166; 10.785) |
| 2 | 2 | 8.934 | 0.788 | (7.125; 10.744) |
| 3 | 2 | 8.85 | 1.59 | (7.04; 10.66) |
| 4 | 2 | 10.70 | 2.45 | (8.90; 12.51) |
| 5 | 2 | 9.518 | 1.213 | (7.709; 11.327) |
| 6 | 2 | 12.296 | 0.595 | (10.487; 14.105) |
| 7 | 2 | 7.4180 | 0.0580 | (5.6089; 9.2271) |
| 8 | 2 | 5.8115 | 0.0615 | (4.0024; 7.6206) |
| 9 | 2 | 9.197 | 0.685 | (7.387; 11.006) |

*Pooled StDev = 1.13098*

**Fisher Pairwise Comparisons**

Grouping Information Using the Fisher LSD Method and 95% Confidence

| TRT | N | Mean | Grouping | | | |
| --- | --- | --- | --- | --- | --- | --- |
| 6 | 2 | 12.296 | A |  |  |  |
| 4 | 2 | 10.70 | A | B |  |  |
| 5 | 2 | 9.518 |  | B | C |  |
| 9 | 2 | 9.197 |  | B | C |  |
| 1 | 2 | 8.976 |  | B | C |  |
| 2 | 2 | 8.934 |  | B | C |  |
| 3 | 2 | 8.85 |  | B | C |  |
| 7 | 2 | 7.4180 |  |  | C | D |
| 8 | 2 | 5.8115 |  |  |  | D |

*Means that do not share a letter are significantly different.*
