# Supplementary material for: Chitosan nanoparticles improve physiological and biochemical responses of Salvia abrotanoides (Kar.) under drought stress
Source: BMC Plant Biol. 2022 Jul 22;22:364. doi: 10.1186/s12870-022-03689-4 (PMC9308334; doi:10.1186/s12870-022-03689-4)
Supplement: Supplementary file 2 — Additional file 2. Some photos of Salvia abrotanoides in the green house and field in Iran. [file 12870_2022_3689_MOESM2_ESM.docx]

**Some photos of *Salvia*** ***abrotanoides* in the green house and field in Iran**


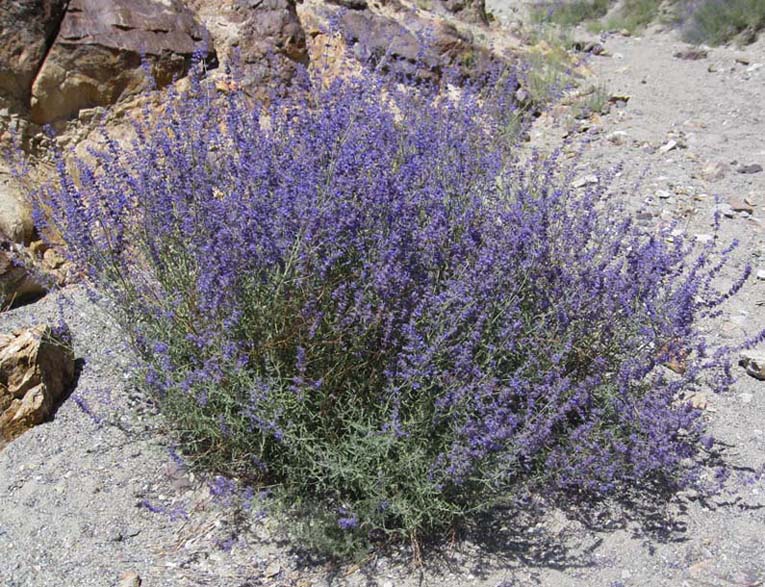

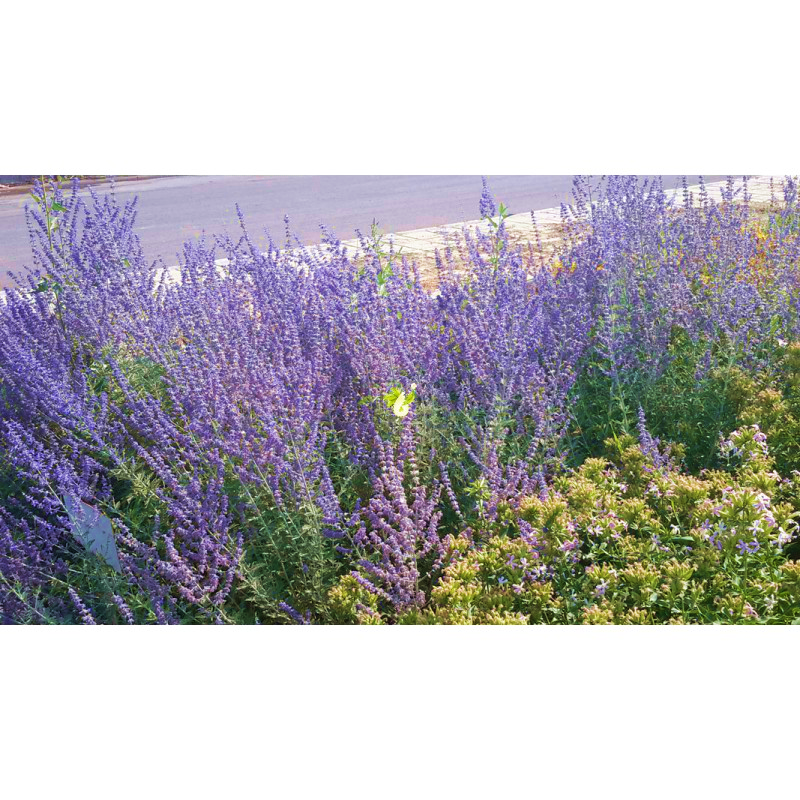
**
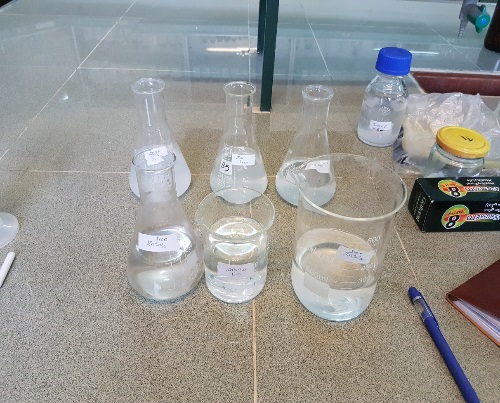
**
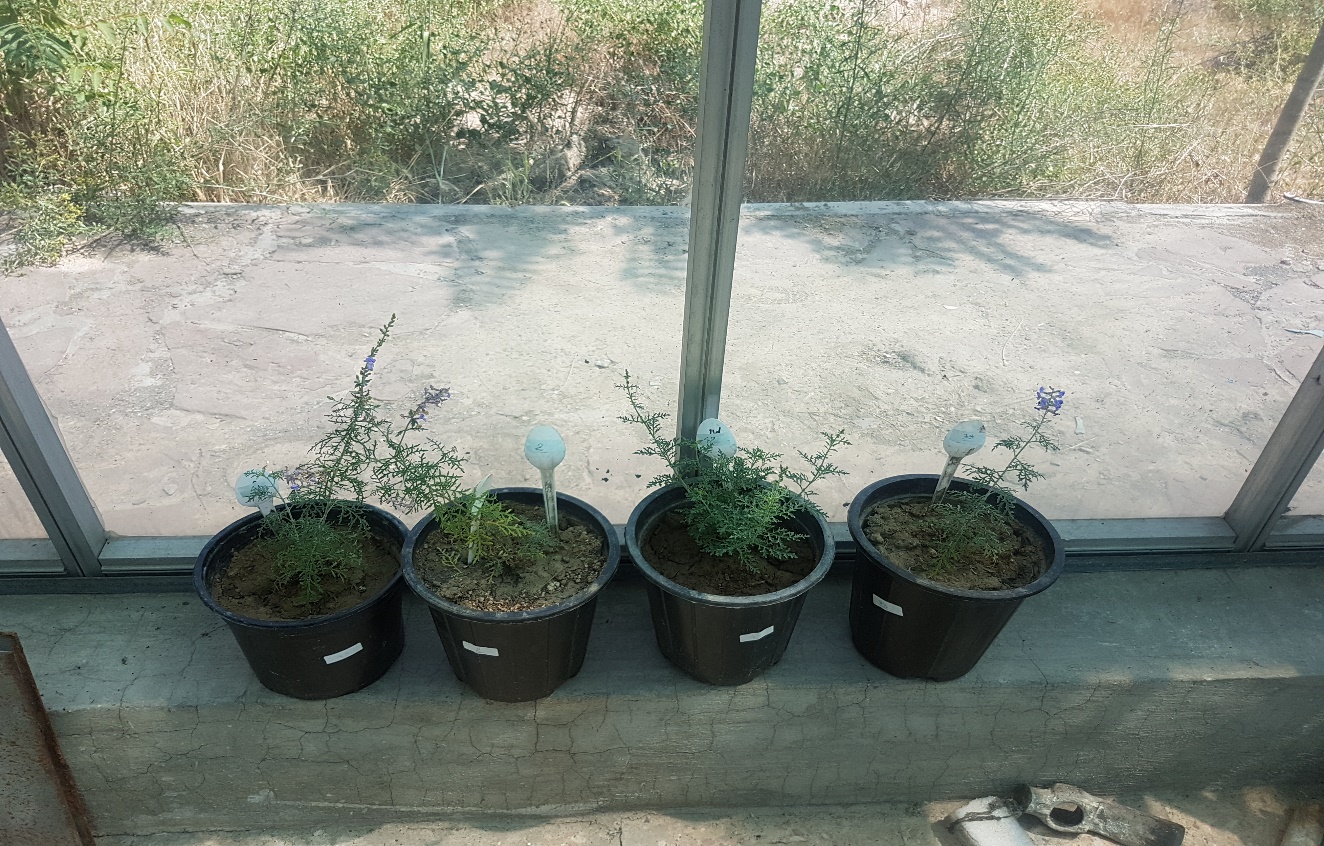

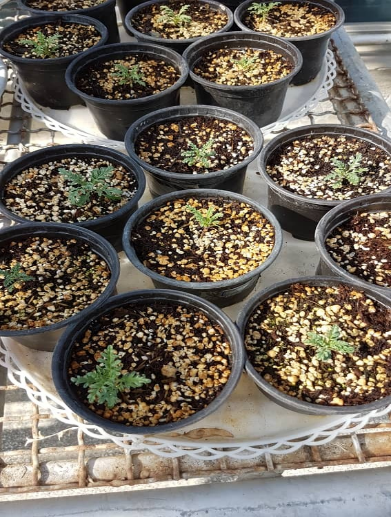

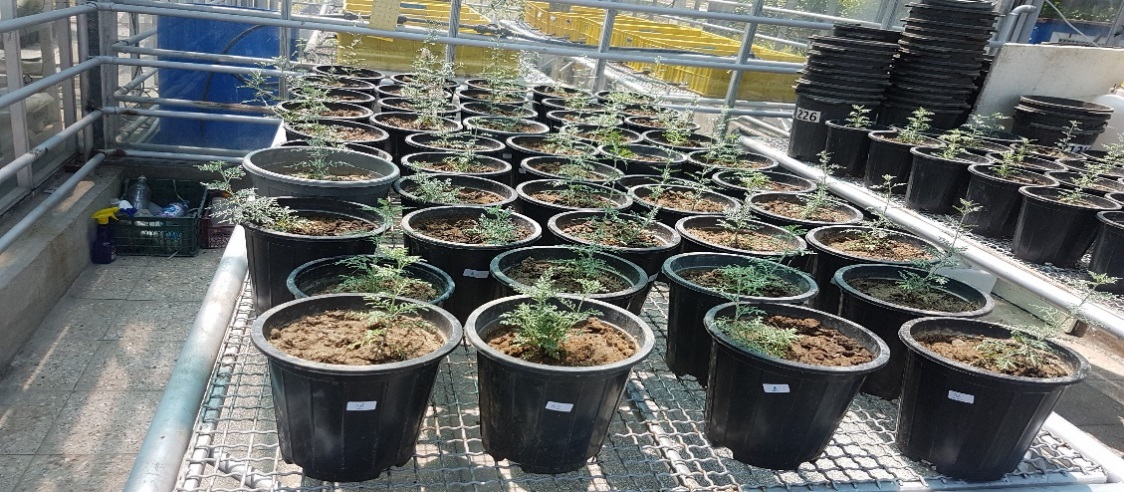

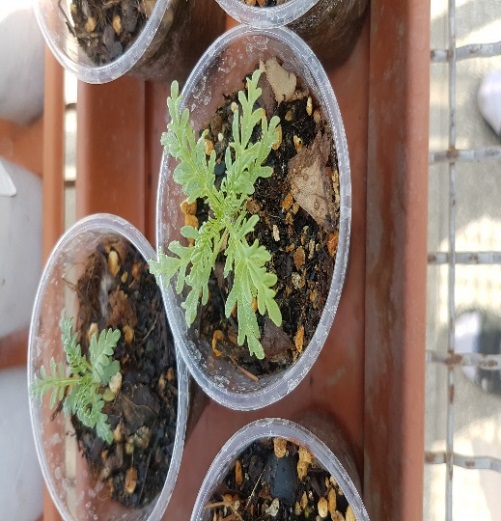


Photos taken of *Salvia abrotanoides (Kar.)* in Research center for plant Sciences, Ferdowsi University of Mashhad, Mashhad, Iran, 2020.

Photos taken of *Salvia abrotanoides* (Kar.) in nature of Iran and in the urban landscape, 2018.
