# Supplementary material for: Chitosan nanoparticles improve physiological and biochemical responses of Salvia abrotanoides (Kar.) under drought stress
Source: BMC Plant Biol. 2022 Jul 22;22:364. doi: 10.1186/s12870-022-03689-4 (PMC9308334; doi:10.1186/s12870-022-03689-4)
Supplement: Supplementary file 3 — Additional file 3: Figure 1. Standard curve of gallic acid. Figure 2. Standard curve of quercetin. Figure 3. Standard curve of glucose. Figure 4. Standard curve of proline. Figure 5. Standard curve of bovine serum albumin (BSA). [file 12870_2022_3689_MOESM3_ESM.docx]

**Standard curves of biochemical parameters**


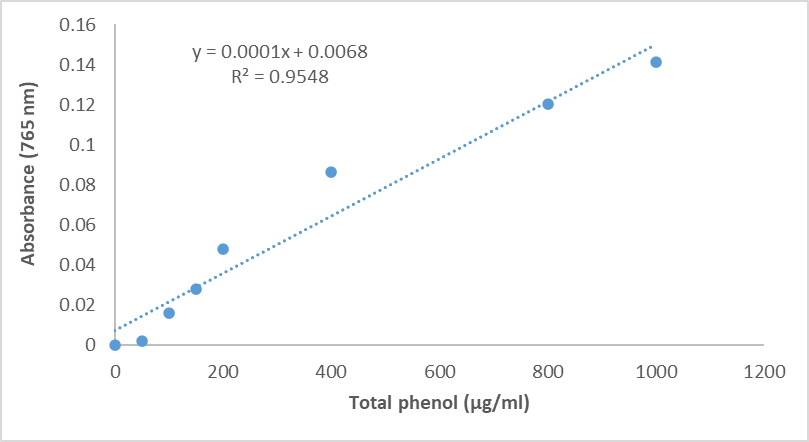


**Fig. 1** Standard curve of gallic acid


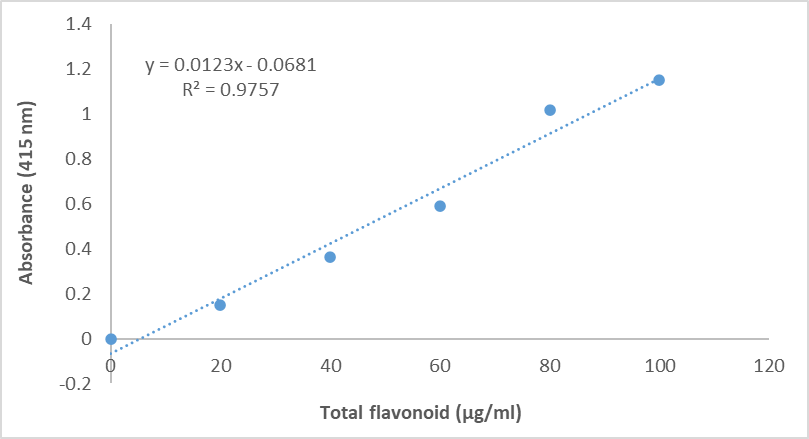


**Fig. 2** Standard curve of quercetin


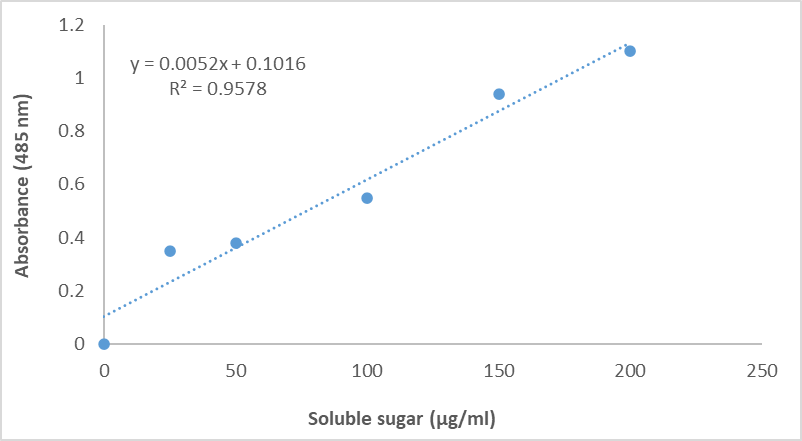


**Fig 3.** Standard curve of glucose


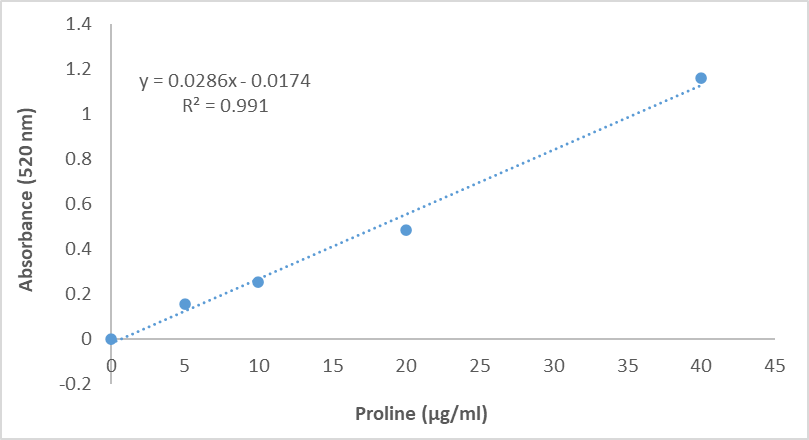


**Fig 4.** Standard curve of proline


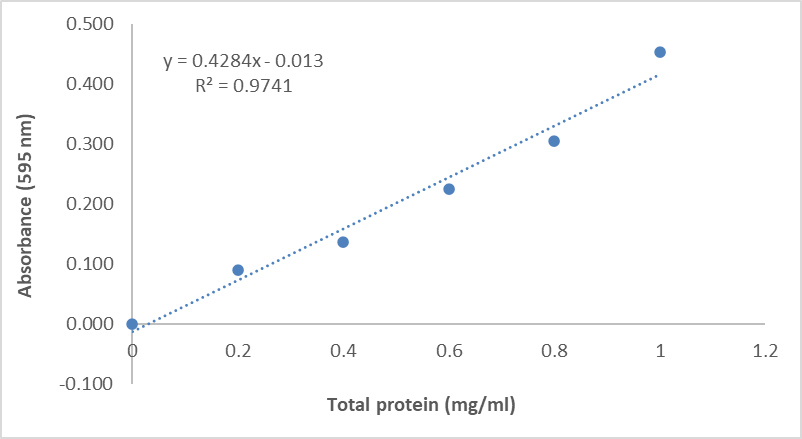


**Fig 5.** Standard curve of bovine serum albumin (BSA)
