# Supplementary material for: Chitosan nanoparticles improve physiological and biochemical responses of Salvia abrotanoides (Kar.) under drought stress
Source: BMC Plant Biol. 2022 Jul 22;22:364. doi: 10.1186/s12870-022-03689-4 (PMC9308334; doi:10.1186/s12870-022-03689-4)
Supplement: Supplementary file 4 — Additional file 4. [file 12870_2022_3689_MOESM4_ESM.docx]

Synthesis of chitosan nanoparticles: The amount of 500 mg of chitosan (medium molecular weight and 85% deacetylated, Sigma Chemical, St. Louis, USA) was dissolved in 50 ml of 1% acetic acid solution and stirred at 1000 rpm for 25 min at room temperature until the solution became clear. The resulting solution was sonicated and then titrated by addition of NaOH or HCL solution adjusted to pH5 and filtered using 0.2µ mesh. For coating process, 5 ml nano-magnetic solution was added to 75 mL deionized water and sonicated for 10 min. Then, chitosan solution was added and sonicated for 5 min. The resulting solution was clear.
