# Supplementary material for: Chitosan nanoparticles improve physiological and biochemical responses of Salvia abrotanoides (Kar.) under drought stress
Source: BMC Plant Biol. 2022 Jul 22;22:364. doi: 10.1186/s12870-022-03689-4 (PMC9308334; doi:10.1186/s12870-022-03689-4)
Supplement: Supplementary file 5 — Additional file 5. [file 12870_2022_3689_MOESM5_ESM.pdf]

Certificate of Analysis

Chitosan Nanoparticles

(C6H11NO4, Purity: >99%, APS: 50nm)
Stock No: NS6130-09-918, CAS: 9012-76-4

Product Name :
Chitosan Nanoparticles

Stock No:	NS6130-09-918
CAS:	9012-76-4
Assay :	>99%
Other Metal:	8000ppm

Note 1: Values are given in % unless otherwise specified.
Note 2: All figures above are weight for weight as determined by ICP

Phone: 05138688890
Mobile: 09129152167
E-mail: nanosany@gmail.com

مشهد-وکیل آباد ٦٤-بنفشه١٢-پلاک٥٠-
طبقه٥-زنگ ٨
