# Supplementary material for: Chitosan nanoparticles improve physiological and biochemical responses of Salvia abrotanoides (Kar.) under drought stress
Source: BMC Plant Biol. 2022 Jul 22;22:364. doi: 10.1186/s12870-022-03689-4 (PMC9308334; doi:10.1186/s12870-022-03689-4)
Supplement: Supplementary file 7 — Additional file 7. [file 12870_2022_3689_MOESM7_ESM.pdf]

Chitosan Nanoparticles

Specification Sheet

$C6H11NO4$

Purity: >99%

APS: 50nm

Stock No: NS6130-09-918

CAS: 9012-76-4

Product: Chitosan Nanoparticles
Stock No: NS6130-09-918
CAS: 9012-76-4
Purity: >99%
APS: 50nm
Molecular Formula: C6H11NO4
Molecular Weight: 161g/mol
Form: Powder
Color: White
Specific Gravity: 1.4
Solubility: Soluble dilute aqueous acid
Main Inspect Verifier: Manager QC

Application:
Chitosan offers a wide range of applications in therapeutic and nonpharmaceutical fields. Chitosan-based nanomaterial have also gained importance in agriculture due to their role in plant growth, physiological responses, nutrient uptake, and antibacterial and antifungal activity in plants. Among various derivatives of chitosan, carboxymethyl chitosan is today the most promising in scientific applications, being used in tissue engineering, drug delivery, microbiology, the cosmetics industry, bioimaging, material sciences, and gene therapy. The chapter highlights various biological and physicochemical properties of carboxymethyl chitosan, and its potential for further development in the near future.

Phone: 05138688890
Mobile: 09129152167
E-mail: nanosany@gmail.com

مشهد-وکیل آباد ٦٤-بنفشه١٢-پلاک٥٠-
طبقه٥-زنگ ٨
