# Supplementary material for: Chitosan nanoparticles improve physiological and biochemical responses of Salvia abrotanoides (Kar.) under drought stress
Source: BMC Plant Biol. 2022 Jul 22;22:364. doi: 10.1186/s12870-022-03689-4 (PMC9308334; doi:10.1186/s12870-022-03689-4)
Supplement: Supplementary file 8 — Additional file 8. [file 12870_2022_3689_MOESM8_ESM.pdf]

MATERIAL SAFETY DATA SHEET

CHITOSAN NANOPARTICLES

Stock #: NS6130-09-918

1. IDENTIFICATION OF THE PRODUCT AND THE COMPANY

Product Name: Chitosan Nanoparticles
Use: Research and Development

2. COMPOSITION & INFORMATION ON INGREDIENTS

Chemical Characterization: $C_6H_{11}NO_4$
Hazardous Ingredients: Nil

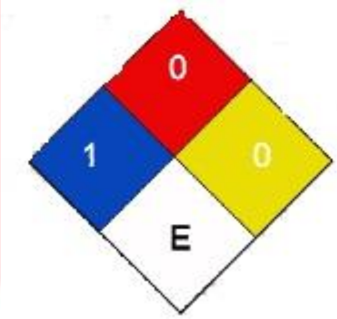

Health	1
Fire	0
Reactivity	0
Personal Protection	E

NANOSANY CORPORATION TM

3. HAZARD IDENTIFICATION

Toxicity: No Data Available
Eye Contact: Dust may cause irritation

4. FIRST AID MEASURES

Skin: Wash skin with soap and copious amounts of water
Eyes: Immediate and prolonged irritation treats with copious amounts of water.
Ingestion: Wash out mouth with water provided person Is Conscious.
Inhalation: If inhaled, remove to fresh air. If not breathing give artificial respiration. If breathing is difficult, give oxygen.

Phone: 05138688890
Mobile: 09129152167
E-mail: nanosany@gmail.com

مشهد-وکیل آباد ٦٤-بنفشه١٢-پلاک٥٠-
طبقه٥-زنگ ٨

5. FIREFIGHTING MEASURES

Extinguishing Data: Water Spray
Unsuitable Extinguishing Data: Carbon Dioxide, Dry Chemical Powder, Polymer Foam
Unusual Firefighting Hazards: Capable of creating a dust explosion Special Firefighting Procedures: Use normal procedures which include Wearing self-contained breathing apparatus and protective clothing to prevent contact with skin and eyes.

6. ACCIDENTAL RELEASE MEASURES

Personal Precautions: Wear respirator, chemical safety goggles, rubber boots and gloves.
Precautions to the Environment: Sweep up, place in a bag and hold for waste disposal.
Cleanup Procedures: Avoid raising dust. Ventilate area and wash spill site after material pickup is complete.

7. HANDLING AND STORAGE

Handling Precautions: Chemical Safety Goggles. Compatible with Chemical-resistant Gloves
Storage: Store in a cool dry place.
Unusable Packaging Materials: Wash thoroughly after handling. Irritating dust, Keep tightly closed

8. EXPOSURE CONTROLS AND PERSONAL PROTECTION

Personal Protective Equipment
Respiratory: Self-contained breathing apparatus
Hand: Chemical-resistant Gloves
Eye: Avoid contact with eyes
Skin: Wash thoroughly after handling

9. PHYSICAL AND CHEMICAL PROPERTIES

NANOSANY CORPORATION TM

Appearance
Form: Powder
Color: White
Odor: No Odour

Safety Related Information
Flashpoint: N/A
Boiling Point: N/A
Melting Point: N/A
PH: N/A

10. STABILITY AND REACTIVITY
Stability: Completely Stable
Reactivity: Non-Reactive/ Non Soluble

11. TOXICOLOGICAL INFORMATION
Possible Health Effects
Skin: No effect
Eyes: Irritation
Inhalation: No Chocking Hazard
Toxicity: Non-Toxic

12. ECOLOGICAL IMPACT
Avoid raising dust. Ventilate area and wash spill site after material pickup is complete.
No Negative Ecological Impact, Data not Available

13. WASTE DISPOSAL
Dissolve or mix the material with a combustible solvent and burn in a chemical Incinerator, equipped with an afterburner and scrubber

14. TRANSPORT INFORMATION (UN ORNEK OLARAK VERİLMİŞTİR)
HS Code: 39139090
CAS: 9012-76-4
Proper Shipping Name: Chitosan Nanoparticles
Air Transport (ICAO & IATA): Clay Nanopowder
Class: Non Hazardous
Packing group: Normal Packing
Transport information: Not regulated for IATA (AIR)

NANOSANY CORPORATION TM

15. OHTER REGULATORY INFORMATION
Federal and State Regulations: TSCA 8(b) inventory: Chitosan Nanoparticles
Other Regulations: EINECS: This product is on the European Inventory of Existing Commercial Chemical Substances.
Other Classifications:
WHMIS (Canada): Not controlled under WHMIS (Canada)
DSCL (EEC): R36- Irritating to eyes
S2- Keep out of the reach of children
S46- If swallowed, seek medical advice immediately & show container or label
HMIS (U.S.A.):
Health Hazard: 1
Fire Hazard: 0
Reactivity: 0

Phone: 05138688890
Mobile: 09129152167
E-mail: nanosany@gmail.com

مشهد-وکیل آباد ٦٤-بنفشه١٢-پلاک٥٠-
طبقه٥-زنگ ٨

Personal Protection: E
National Fire Protection Association (U.S.A.):
Health: 1
Flammability: 0
Reactivity: 0
Specific hazard:
Protective Equipment: Gloves. Lab coat.
Dust respirator. Be sure to use an approved/certified respirator or equivalent.
Splash goggles.

16. OTHER INFORMATION

References: Not available
Other Special Considerations: Not available
Date of Print: May 9, 2019

NANOSANY CORPORATION TM

Phone: 05138688890
Mobile: 09129152167
E-mail: nanosany@gmail.com

مشهد-وکیل آباد ٦٤-بنفشه١٢-پلاک٥٠-
طبقه٥-زنگ ٨
