# Supplementary figures and images for: Chitosan nanoparticles improve physiological and biochemical responses of Salvia abrotanoides (Kar.) under drought stress
Source: BMC Plant Biol. 2022 Jul 22;22:364. doi: 10.1186/s12870-022-03689-4 (PMC9308334; doi:10.1186/s12870-022-03689-4)

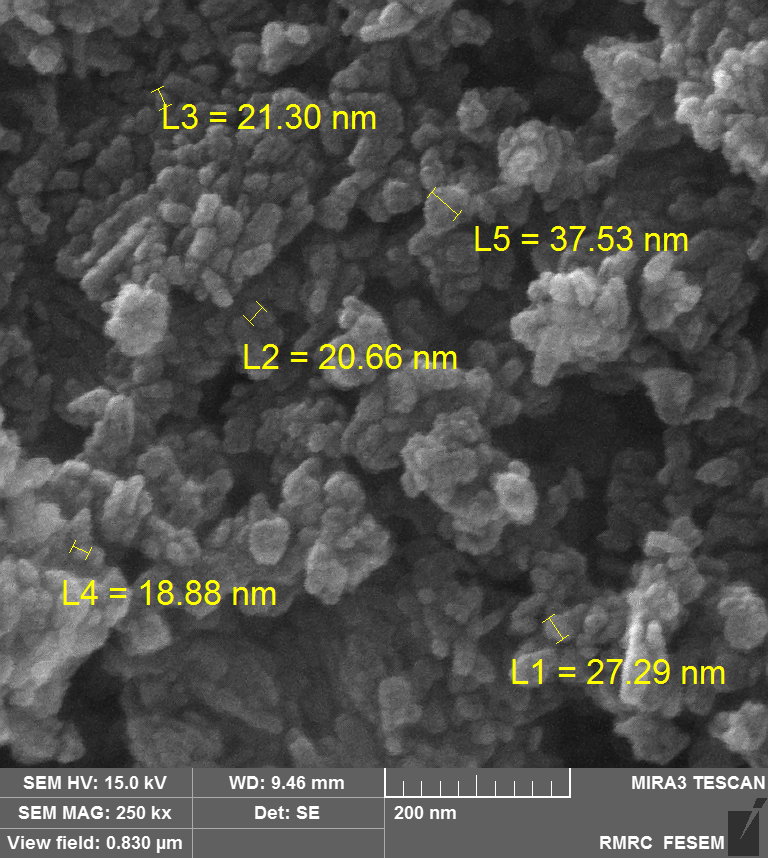

Supplement: Supplementary file 10 — Additional file 10. [file 12870_2022_3689_MOESM10_ESM.tif]

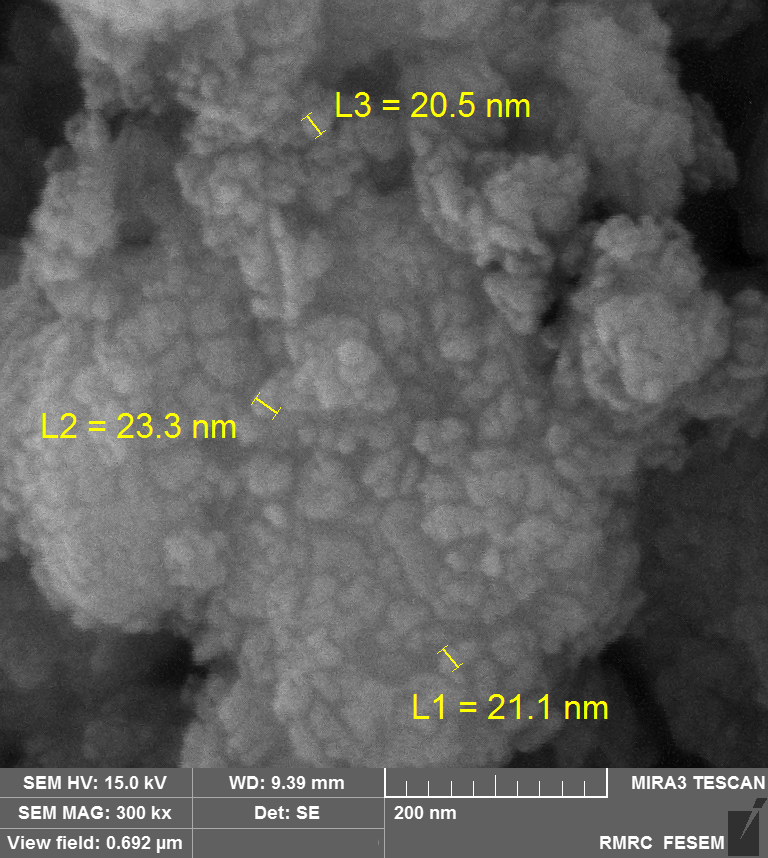

Supplement: Supplementary file 11 — Additional file 11. [file 12870_2022_3689_MOESM11_ESM.tif]

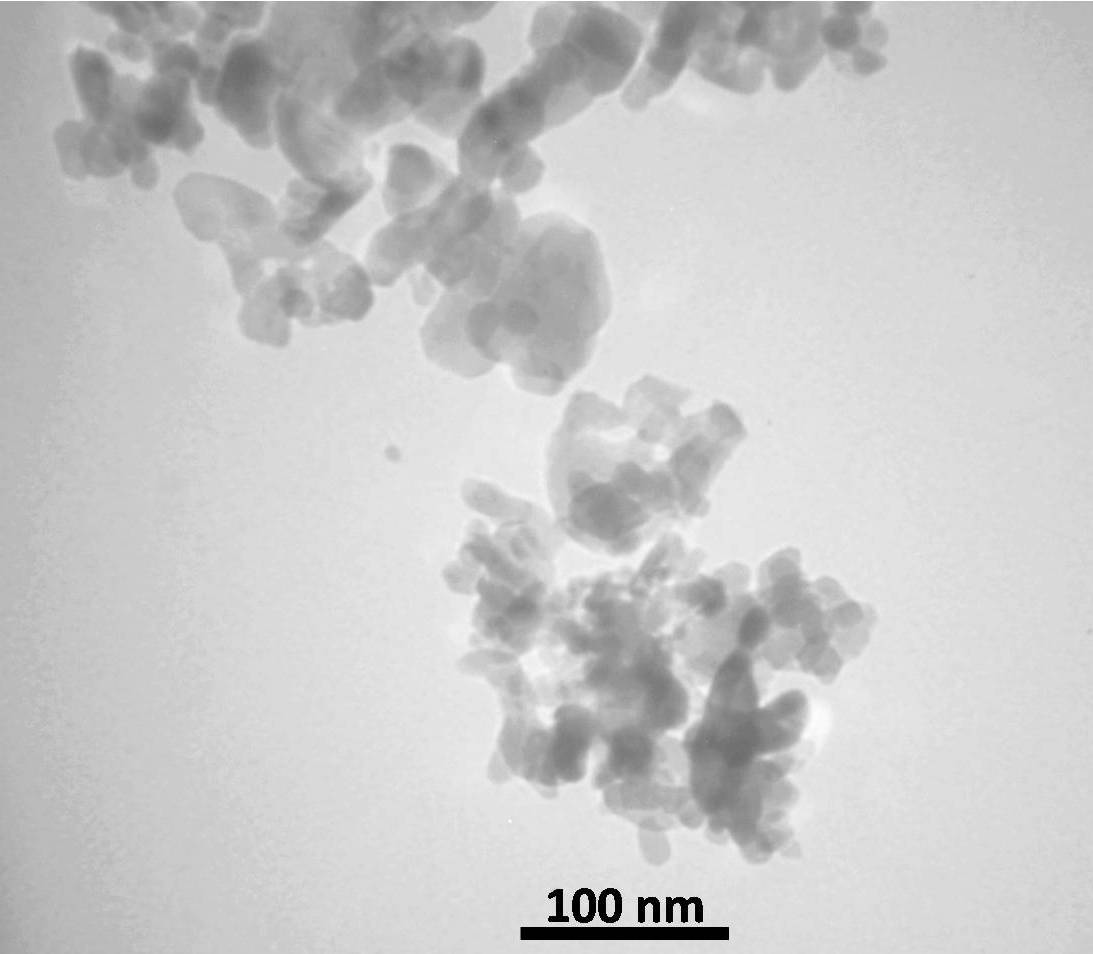

Supplement: Supplementary file 12 — Additional file 12. [file 12870_2022_3689_MOESM12_ESM.jpg]

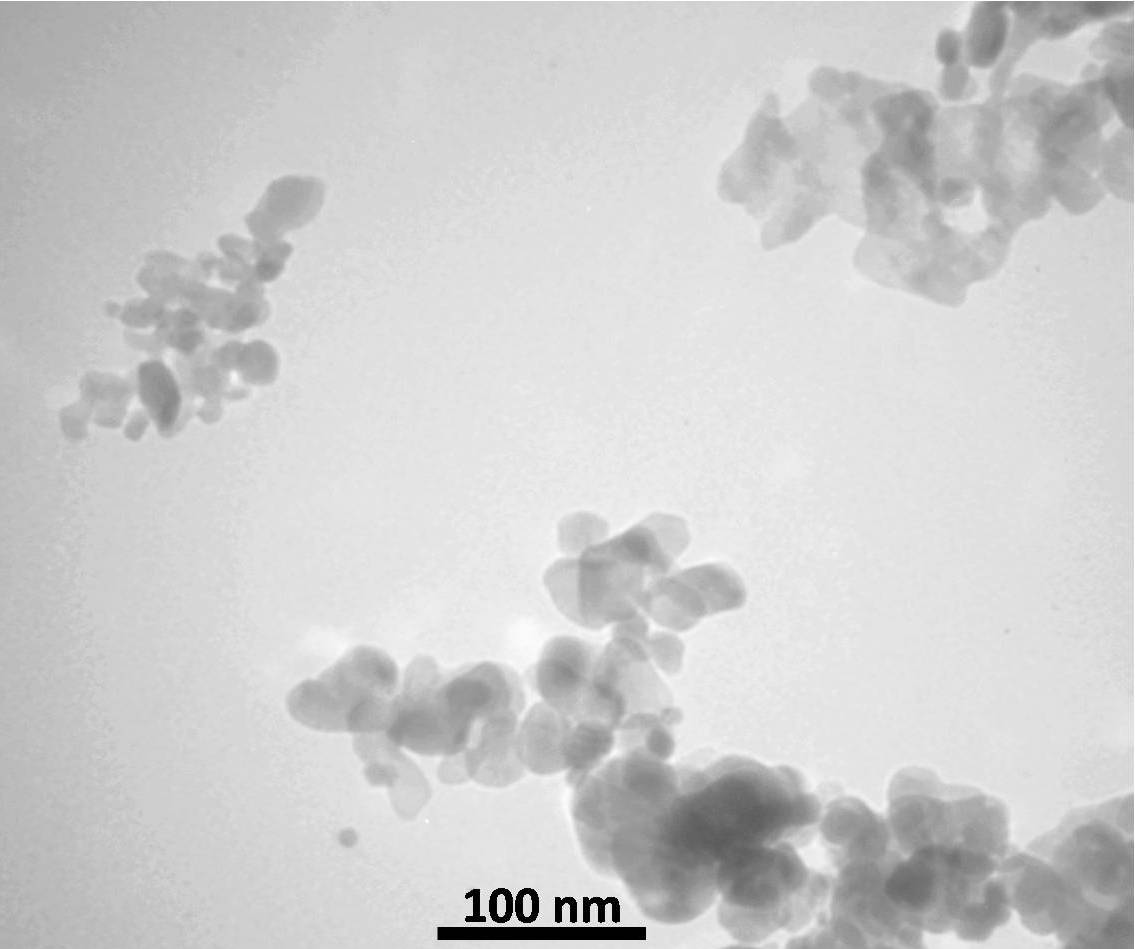

Supplement: Supplementary file 13 — Additional file 13. [file 12870_2022_3689_MOESM13_ESM.jpg]

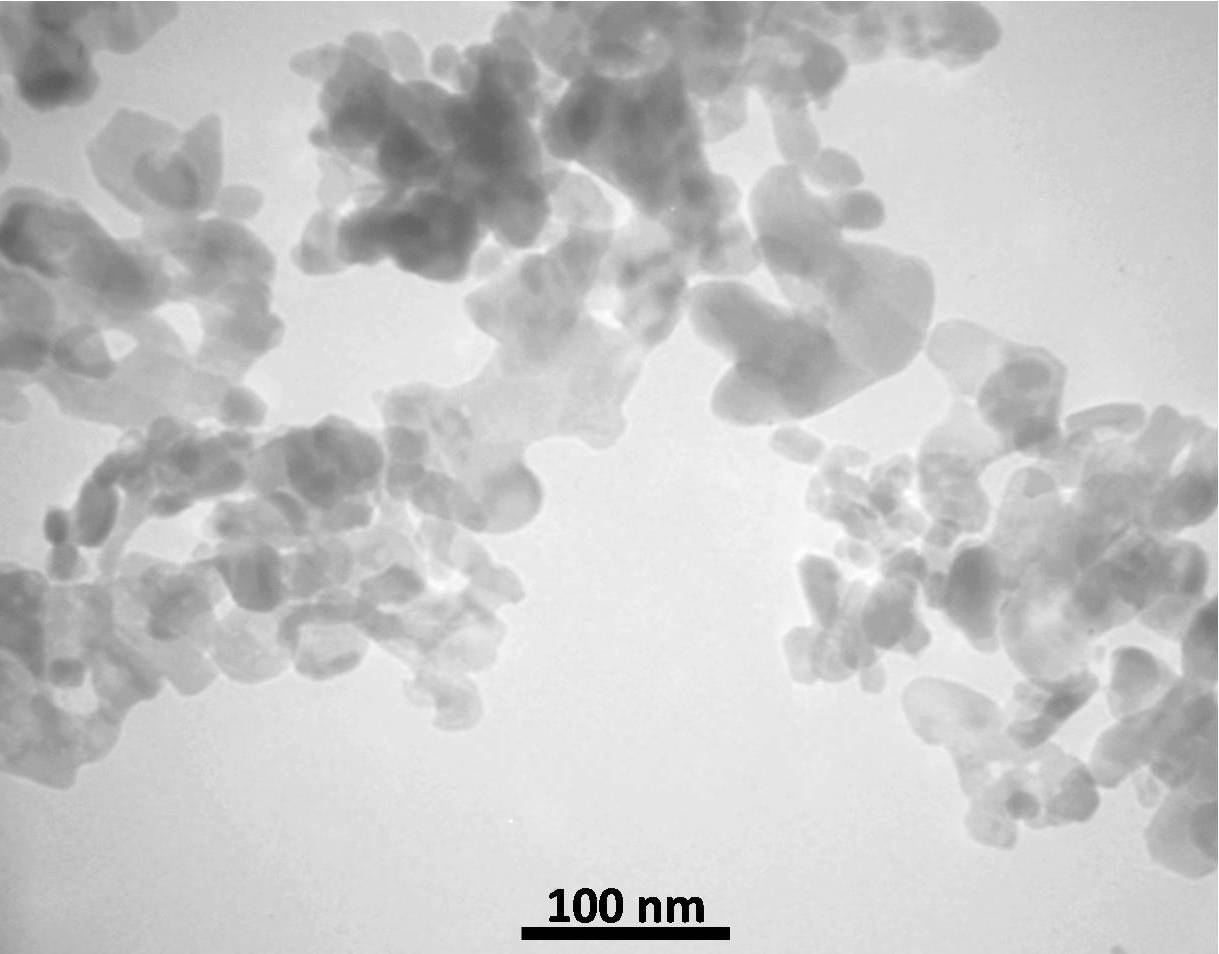

Supplement: Supplementary file 14 — Additional file 14. [file 12870_2022_3689_MOESM14_ESM.jpg]

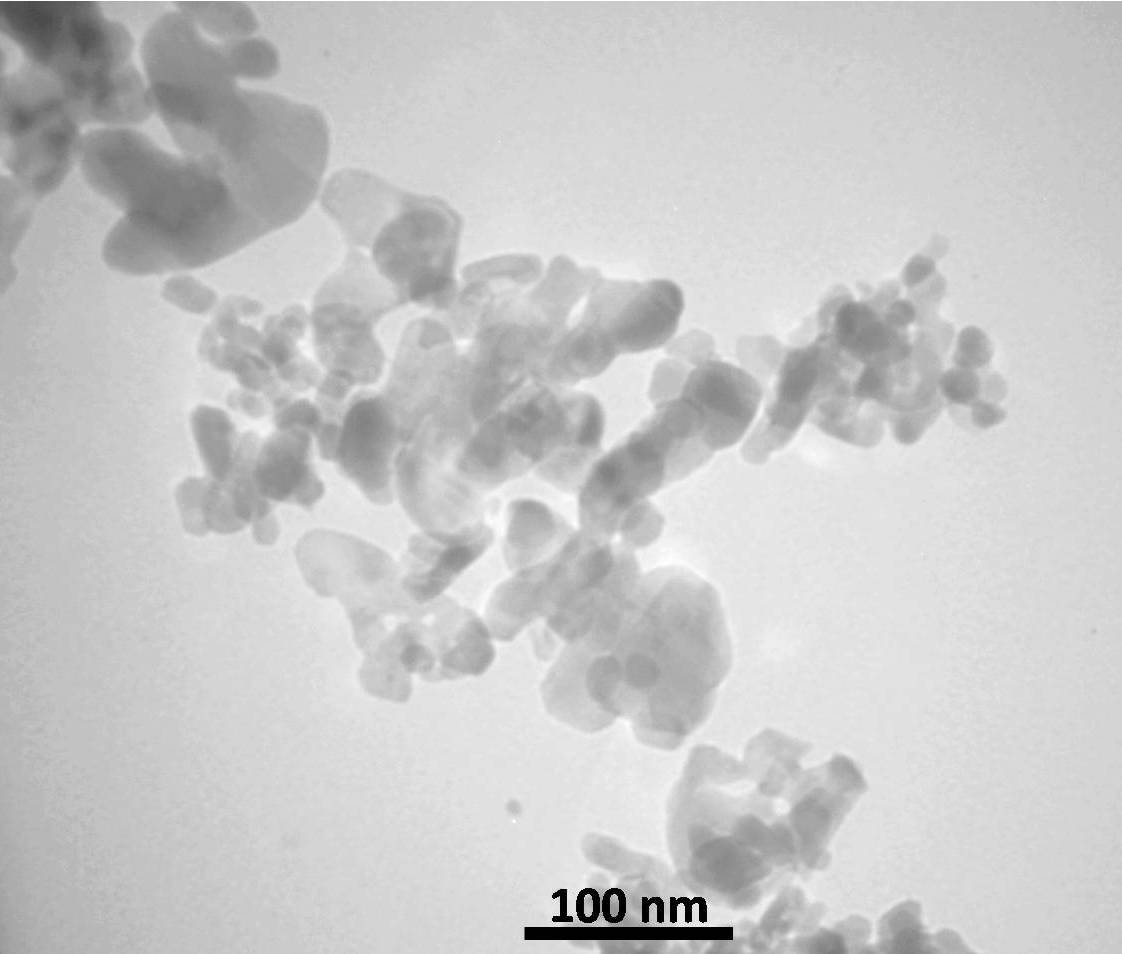

Supplement: Supplementary file 15 — Additional file 15. [file 12870_2022_3689_MOESM15_ESM.jpg]
